# Computational modeling of lignin: from molecular structure to functional properties

**DOI:** 10.1039/d6ra04854d

**Published:** 2026-08-20

**Authors:** Raju Kumar, Igor Zozoulenko, Aleksandar Y. Mehandzhiyski

**Affiliations:** a Laboratory of Organic Electronics, Department of Science and Technology (ITN), Linköping University Campus Norrköping 60174 Norrköping Sweden aleksandar.mehandzhiyski@liu.se; b Wallenberg Wood Science Center, Linköping University SE-601 74 Norrköping Sweden

## Abstract

Lignin is one of the abundant aromatic biopolymers in nature, constituting approximately 15–30% of the lignocellulosic biomass and representing a major renewable source of aromatic carbon. Its high carbon content, high thermal stability, biodegradability, antioxidant property, and ability to absorb ultraviolet radiation make lignin an attractive precursor for many potential applications, such as for renewable carbon materials, plastics, additives, surfactants, and the resin industry. However, its large-scale valorisation remains limited due to its complex, heterogeneous structure and the limited ability of experimental techniques to resolve its molecular architecture and reaction mechanisms. Recent advancements in computer-based simulations, especially molecular dynamics (MD) and density functional theory (DFT), offer powerful tools to overcome these limitations by enabling molecular and atomic-level insight into lignin structure, properties, and transformation processes. These methods provide detailed insights into lignin reactivity, thermophysical behaviour, self-assembly, and catalytic depolymerisation that are often difficult to obtain experimentally. This review summarises recent advances in computational studies of lignin and its derivatives, highlighting how different modelling approaches have been used to investigate the various physicochemical properties and reaction mechanisms. In addition, the limitations and challenges associated with current modelling strategies are discussed, and future research directions, including multiscale modelling and machine-learning-based approaches, are outlined. Overall, this review aims to provide a comprehensive overview of how computational methods advance lignin research and support the development of sustainable lignin-based materials.

## Abstract 2

Computational simulations and modeling accelerate lignin valorisation, by revealing its structure, reactivity and functional properties.

## Graphical abstract

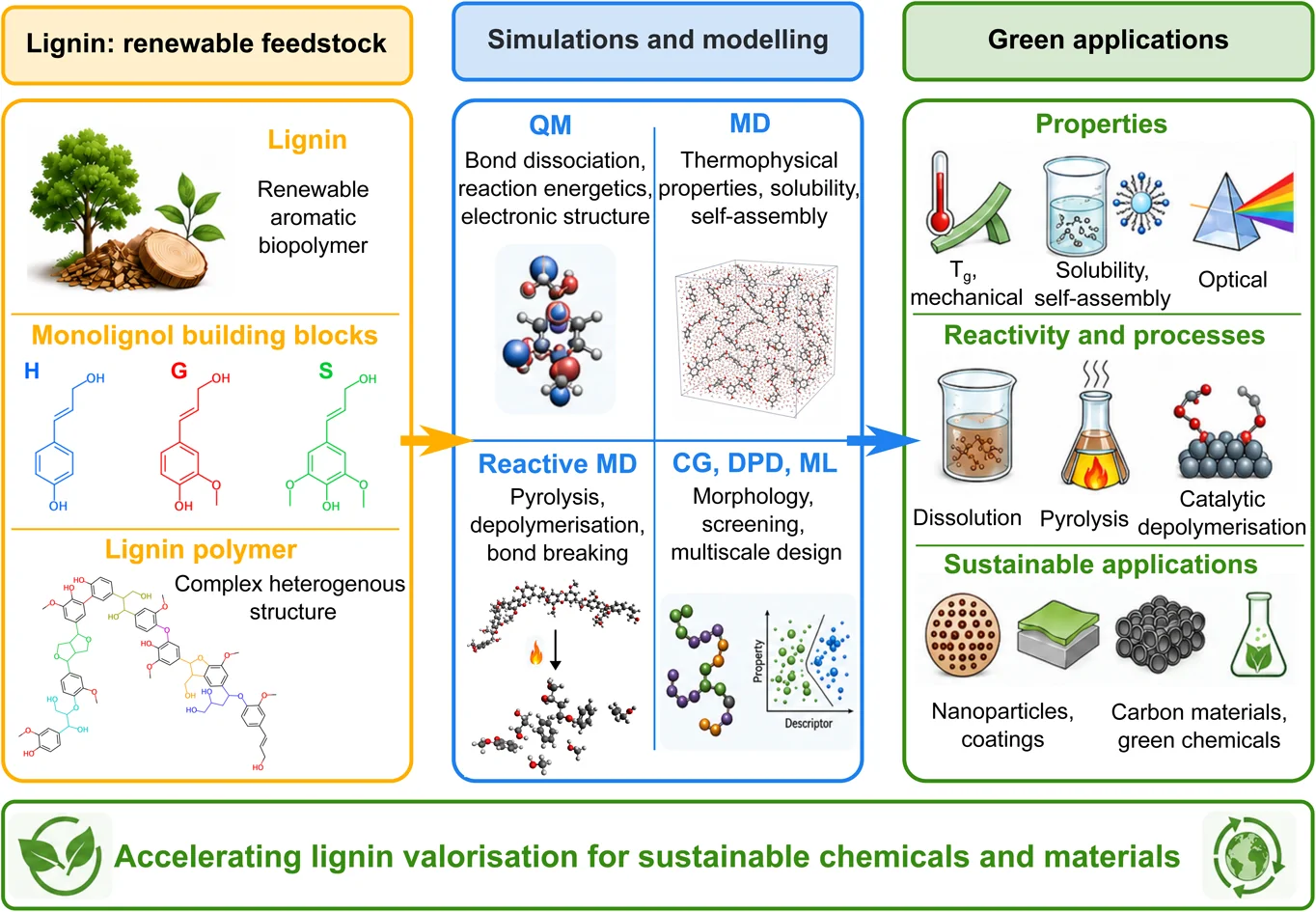

## 1 Introduction

Rapid global population growth has increased material and energy demands, resulting in a significant impact on sustainability and the environment in various ways. At present, these demands rely predominantly on fossil-based raw materials, particularly crude oil-derived polymers, which have contributed substantially to plastic pollution, greenhouse gas emissions, and depletion of natural resources. To address these challenges, there is an urgent need to develop renewable, sustainable materials that reduce the environmental impact of man-made materials while meeting current and future social needs.^1–3^

**Fig. 1:**
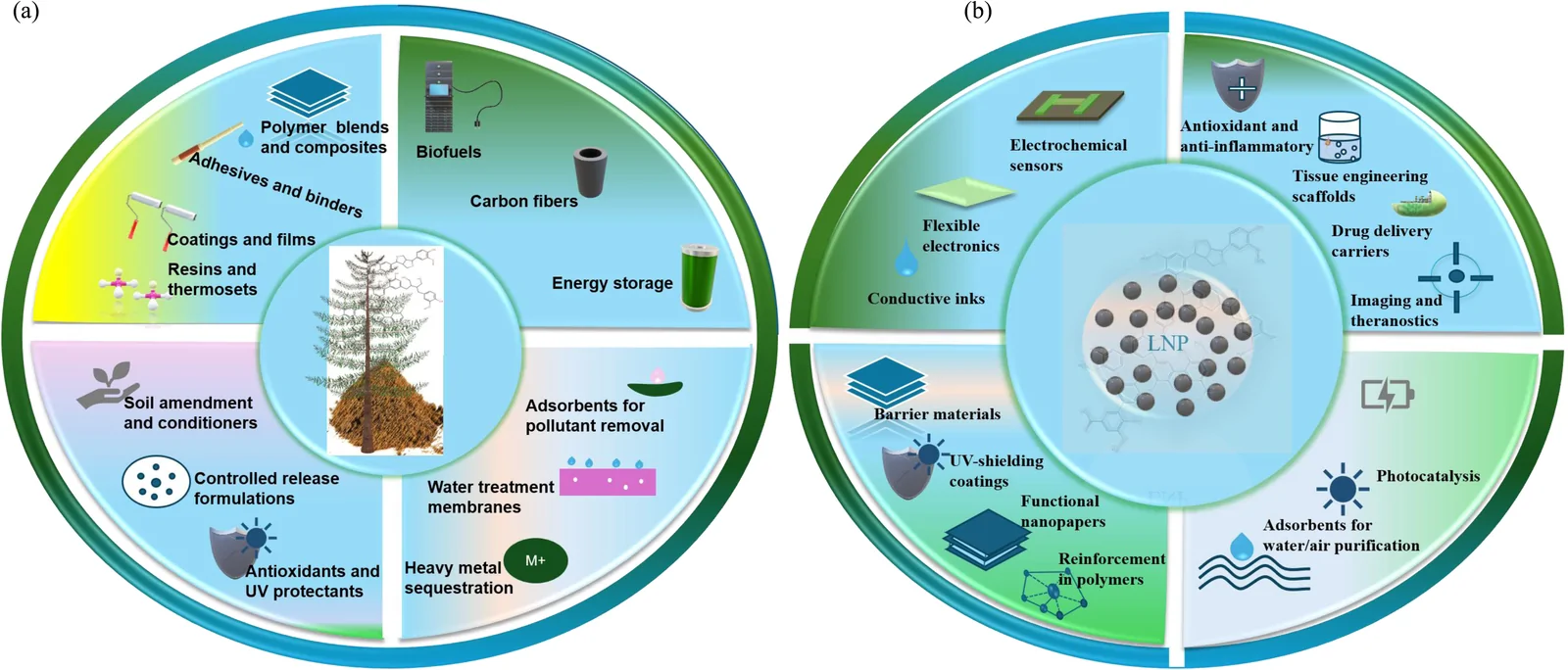
Applications of (a) lignin and (b) lignin nanoparticles (LNPs).

Plant biomass is one of the resources that could significantly contribute to solving global plastic pollution, climate, and environmental challenges associated with materials usage.^1,4^ Plant biomass primarily originates from agriculture, grasses, and various forest plants, which are mainly composed of cellulose, hemicellulose, and lignin,^5^ and is often called lignocellulosic biomass. Lignocellulosic-based biomass has shown great potential as a renewable source to fulfil the demand for sustainable biofuels, chemicals, and materials. Lignin is the most abundant natural source of aromatic compounds and can serve as a chemical feedstock for various applications, as shown in Fig. 1(a), including renewable carbon materials, plastics, additives, surfactants, and resins^6^ thus, substituting petrochemicals. Recently, to expand the horizons of lignin applications, researchers have proposed modifying lignin to produce lignin nanoparticles (LNP), which could be used in various applications, such as drug delivery systems, coatings and paints, biocatalysts, nanocomposite materials, heavy-metal ion adsorption, and more,^7–10^ as shown in Fig. 1(b). The details of the formation of LNPs and their processing methods can be found in previously reported articles.^10,11^ Since these nanoparticles are derived from lignin, they are sustainable, eco-friendly and biodegradable compared to widely used synthetic nanoparticles such as polystyrene particles. These LNPs exhibit greater antioxidant activity than their parental biopolymer due to their high surface-to-volume ratio.^12^ Therefore, LNPs have great potential to become the next generation of sustainable particles, thus substituting synthetic particles, which contribute to pollution and the spread of micro- and nanoplastics throughout all of Earth's subsystems.^13^

Approximately 15–30% of the lignocellulosic biomass is lignin, and 300 billion tonnes are available in the biosphere, of which 50 to 70 million tonnes of lignin are produced from the paper and pulp industry.^14^ However, despite lignin being abundant in nature, the application of lignin-based materials is still in its infancy stage due to the recalcitrance and chemical complexity of lignin structures.^5^ Additionally, our understanding of lignin remains limited, particularly regarding the relationships between isolation, structure, and properties, which are crucial for safe and successful industrial utilisation in material applications.^15^ As a result, lignin utilisation for various value-added functional materials and products is negligible (∼2%), and it is mostly burned to recover pulping chemicals and generate heat and electricity.^16^ This represents an enormous waste of this natural resource and apparently makes this material one of the most abandoned and least efficiently utilized materials on Earth. Moreover, the valorisation of lignin remains underutilised due to the limitations of traditional experimental methods in providing detailed insight into biomass conversion processes, including oxidation, depolymerisation, pyrolysis, and catalytic depolymerisation. These experiments often have limited ability to provide real-time analysis of lignin structure due to resolution limitations and dynamic changes in molecular structure induced by temperature. To go beyond today's limited utilization of lignin, understanding lignin's degradation, synthesis, nanoparticle formation, reaction mechanisms, and structure–property relationships is essential for developing renewable and sustainable functional materials. However, most previous works have been experimental or based on old-fashioned “trial-and-error” empirical approaches, lacking solid theoretical support and detailed insight into these properties. Apart from these, computational approaches have also been employed to other components of plant biomass, particularly cellulose^17,18^ and hemicellulose^19,20^ due to their regular and well-defined structures. These approaches have also been applied to alternative bio-based materials such as chitin^21^ to explore their possibility as alternative renewable materials. But despite advances in computational approaches, theoretical studies devoted to the molecular modelling of lignin remain very limited. As a result, most fundamental questions of vital importance for lignin chemistry and material development remain poorly understood and are not addressed theoretically.

**Fig. 2:**
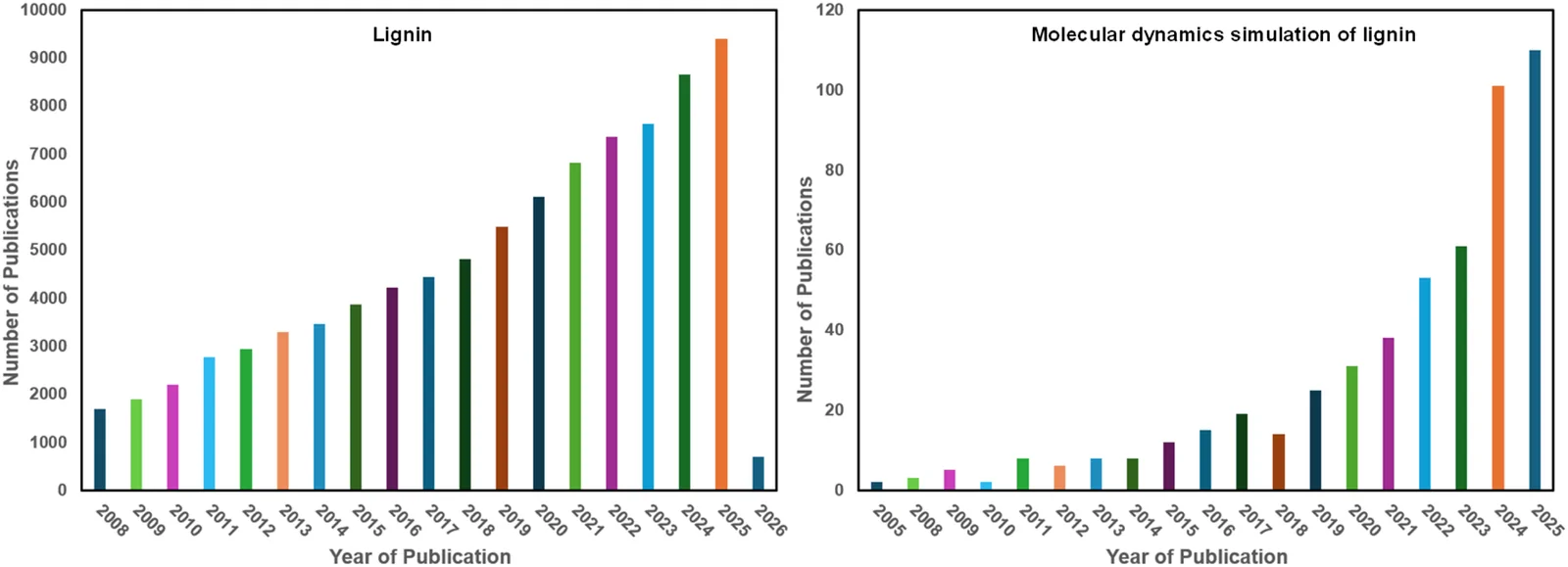
Number of publications based on lignin (left panel) and molecular dynamic simulation of lignin (right panel), according to ISI Web of Science (data obtained January 2026).

Due to advancements in molecular modelling and simulations over the past few decades, computational techniques can provide valuable insights into predicting various properties and offer explanations for experimentally observed thermodynamic, molecular, and macroscopic lignin properties. Recently, due to advancements in computing power and computational algorithms, simulations are emerging as a mainstream direction in materials research, providing essential insights into time- and spatial-scales that are not accessible experimentally and significantly enhancing the efficiency of material design.^4,22^ Researchers often apply a combination of experimental, computational, and analytical approaches to overcome these limitations and provide a more comprehensive study of lignin behaviour. Many researchers have used various electronic structure methods, such as density functional theory (DFT), to investigate the properties of lignin-based biomass. These DFT calculations are mostly used for lignin monomers, dimers, and short oligomers with several tens to a few hundred atoms and provide invaluable insight into the electronic structure of lignin and can predict, for example, the products of lignin pyrolysis.^23–26^ These DFT-based simulations are accurate but less cost-effective than molecular dynamics (MD) simulations for handling large atomic systems. Whereas MD-based simulations can simulate larger systems having approximately 10^3^–10^6^ atoms over timescales ranging from nanoseconds to microseconds, allowing the investigation of lignin conformation, solvation, aggregation, diffusion and morphological organisation. The accuracy of any MD-based simulation also depends on the force field employed, and current modelling approaches are limited by the lignin chemistry covered by the available force fields. Earlier, researchers have explored the basic properties of lignin using atomistic simulations,^5,6,27^ but they have primarily focused on small molecular models, typically ranging from individual oligomers to a limited number of lignin macromolecules and used shorter time intervals, which are insufficient to accurately reproduce experimentally determined lignin properties. However, developing specific force–field parameters for lignin consistent with the CHARMM^28^ and AMBER^29^ force fields enables researchers to model lignin structure across a broad range of environments, leading to a better understanding of its chemical and structural properties.^6,30^ The interaction of lignin with cellulose surfaces^31,32^ the conformational and intramolecular hydrogen-bonding description of monolignols, and the effect of pretreatment on biomass^33^ are a few examples that highlight the use of MD simulations in lignin research. These computational-based studies have provided insight into lignin aggregation behaviour and its interaction with cellulose, hemicellulose, solvents, and enzymes. Among the available computational approaches, MD and DFT have gained widespread attention for characterising biomass at the molecular level due to their excellent ability to reproduce experimental data as evident from the number of publication trends plotted in the Fig. 2. These computationally based approaches can provide insight into the accurate structures, energies, and molecular properties of lignocellulosic biomass that are difficult or even impossible to obtain experimentally.

Several reviews have discussed computational modelling of lignocellulosic biomass and biomass conversion processes, including multiscale simulations of plant cell walls and biorefinery reactions. For example, Zhou *et al.*^1^ reviewed computational modelling approaches for plant biomass components including cellulose and lignin, while Ciesielski *et al.*^4^ highlighted multiscale modelling strategies for lignocellulosic biomass systems. Earlier work by Sangha *et al.*^5^ summarized molecular simulation studies of lignin, and Kong and Fu^34^ reviewed computational approaches for biomass conversion processes. Khodayari *et al.*^35^ offered a review on molecular dynamics simulations in advancing the understanding of the physico–chemical properties of plant cell wall modelling providing atomistic insights into cellulose, disordered cellulose, and hemicelluloses. However, these studies either focus broadly on lignocellulosic biomass or emphasize specific processes such as biomass conversion or dissolution plant cell wall structure. A comprehensive review that systematically summarizes computational investigations of lignin properties and behaviour across different modelling approaches, including DFT, molecular dynamics, reactive simulations, and machine learning methods, remains lacking. Therefore, in this review article we summarise the current state of the art in computational studies of lignin, focusing on how different modelling approaches including DFT, MD, reactive MD simulations, and machine learning methods have been applied to investigate lignin structure, properties, and reactivity. In addition to reviewing recent advances, we critically discuss the limitations and challenges associated with modelling such structurally complex and heterogeneous biopolymers. Finally, we outline key perspectives for future research, highlighting open challenges in lignin modelling and identifying opportunities for the development of improved simulation methodologies, multiscale approaches, and data-driven models that can further advance our understanding and utilization of lignin-based materials.

This review article is divided into four sections. Section 1 is the Introduction. Section 2 discusses the various structures and types of lignin, whereas Section 3 provides insight into lignin and its derivatives through different computational approaches. Section 4 outlines the future perspectives and outlook.

## 2 Structure and chemical diversity of lignin

**Fig. 3:**
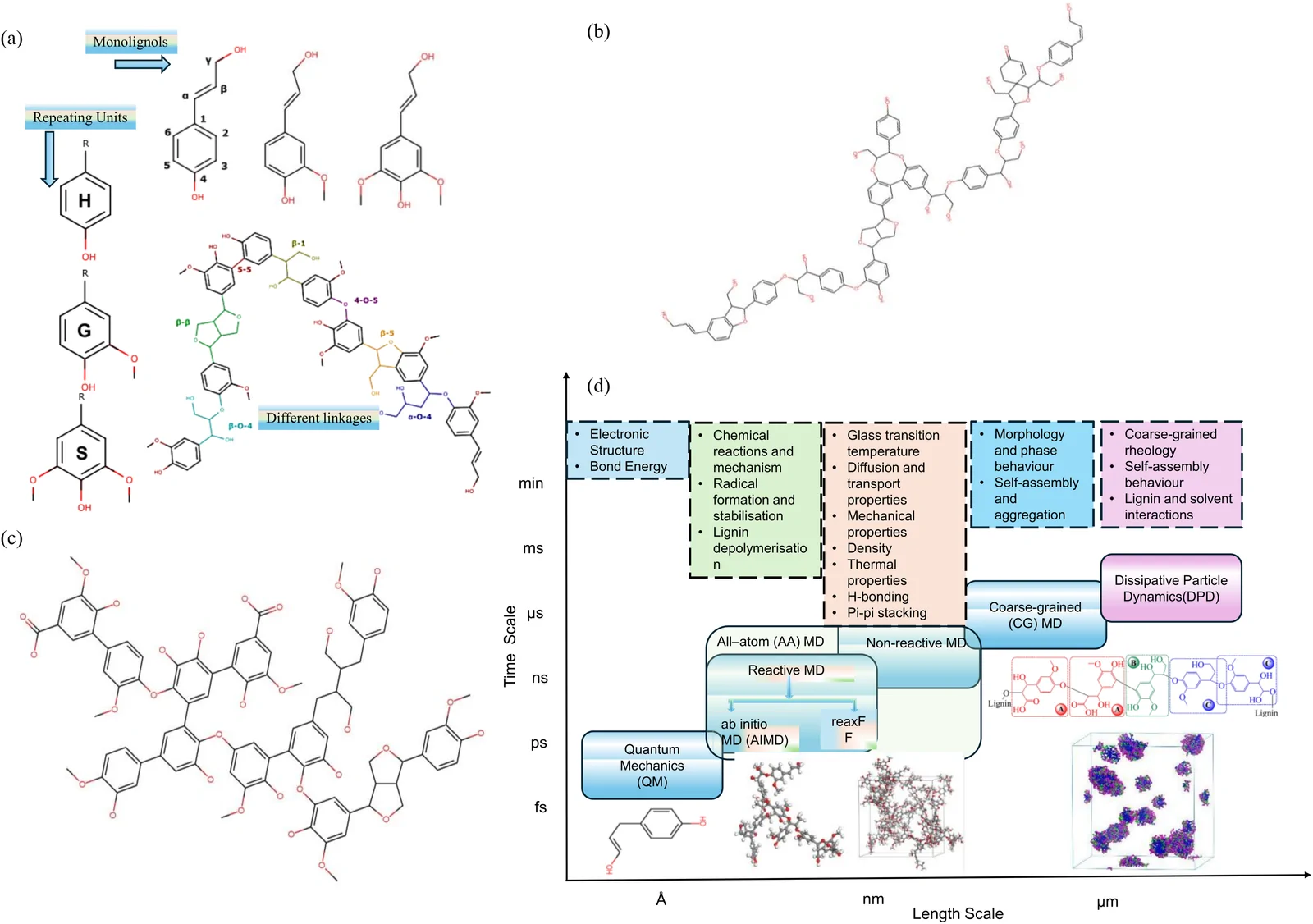
Chemical structures of (a) the different monolignols, repeating unit monomers and linkages in lignin (b) native lignin (this figure has been reproduced from ref. 41 from the Royal Society of Chemistry, copyright 2018) (c) technical lignin (Kraft lignin) (this figure has been reproduced from ref. 42 from the Royal Society of Chemistry, copyright 2017) (d) computational methods and properties for different time and length scales. (Snapshots in Fig. 3(d) have been reproduced from ref. 43 (under the terms of the Creative Commons Attribution-Non-commercial 4.0 License, from Nature, copyright 2024) and ref. from ref. 44 from Springer Nature, copyright 2023).

Lignin is the most abundant heteroaromatic polymer in the plant cell, formed by radical coupling chemistry. Its composition and topology may vary based on the biomass source.^1,36,37^ It is composed of the three fundamental phenylpropanoid precursors, namely *p*-coumaryl alcohol, coniferyl alcohol, and sinapyl alcohol, which are collectively referred to as monolignols. These precursor molecules are transported to the plant cell wall and oxidised enzymatically to form phenoxy radicals. The resulting radicals undergo non-template-driven coupling reactions and make a heterogeneous lignin network containing several interunit linkages, such as β-O-4, β–β, β-5, β-1, 4-O-5, α-O-4, and 5–5 (as shown in Fig. 3(a)). The term “monomer” refers to the free monolignol precursor before polymerisation, whereas the corresponding residues after incorporation into lignin are described as structural units. Thus, *p*-coumaryl alcohol gives rise mainly to *p*-hydroxyphenyl units, coniferyl alcohol to guaiacyl units, and sinapyl alcohol to syringyl units, normally abbreviated as H, G, and S, respectively (as shown in Fig. 3(a)). With the combination of these three monomers, there are several different linkages possible, in which the β-O-4 (alkyl aryl ether) linkage is the most abundant linkage in native lignin.^6^ The different linkages in lignin have different bond dissociation energies (BDE),^6^ which leads to different relative stability for various lignin configurations depending on the bond distribution.

Lignin provides rigidity and mechanical strength of the plant cell wall, facilitates water conduction through the plant stem, and prevents the cell wall from microbial, enzymatic, and chemical degradation.^14^ In general, it is classified into two broad categories, *i.e.*, natural lignin (Fig. 3(b)) and technical lignin (Fig. 3(c)). Natural lignin is found in plants, while technical lignin is the product of the pulping and bleaching processes in the pulp and paper industry.^38,39^ Natural lignin is further classified into softwood (rich in G units), hardwood (containing both G and S units) and herbaceous lignin (containing G, S, and H units). During the harsh chemical and physical processes in the pulping industry, the structure of native lignin is heavily modified, and the structure and linkage types of technical lignin may differ significantly from those of native lignin. Technical lignin is broadly classified into kraft lignin, lignosulfonates, soda lignin and organosolv lignin.^40^ The structure of lignin may vary depending on the extraction method and the plant from which it has been derived. Moreover, technical lignin often contains recalcitrant carbon–carbon linkages that are difficult to break, thereby limiting its tailored applications. As a result, most technical lignin generated from the pulp and paper industry is primarily used for heat and energy rather than advanced materials applications.^38^ In summary, due to its complex and heterogeneous structure, its valorisation for different applications remains limited. Additionally, experimental characterisation tools alone will not be able to specify the structure–function relationship in such cases.

## 3 Computational studies of lignin properties

Computational modelling has become an important tool for understanding lignin at the molecular level. In this section, we review how methods such as DFT and MD simulations have been applied to investigate the thermophysical, mechanical, solubility, electronic, and reactive properties of lignin.

### 3.1 Thermo-physical and mechanical properties

The processability and end-use performance of lignin strongly depend on its thermo-physical properties and behaviour,^45^ which in turn are determined by lignin's chemical structure and molecular weight. Due to lignin's complex molecular structure and structural heterogeneity (variable linkage distributions, degrees of crosslinking, and multiple monomers), determining its thermo-physical properties is inherently challenging. Therefore, combining experimental measurements with molecular simulations provides valuable insights into these properties at the molecular level.

#### 3.1.1 Glass transition temperature (*T*_g_)

The glass transition temperature (*T*_g_) is an intrinsic property of polymers and amorphous materials, indicating the temperature at which they transition from a glassy to a rubbery state, and it depends on the material's structural composition and mechanical properties. Due to lignin's aromatic backbone and a 3D network of phenylpropanoid units, lignin exhibits a high and variable glass transition temperature.^46^ Generally, the glass transition temperature of lignin mainly depends upon the chain flexibility, molecular weight, cross-linking, geometrical factors, branching, crystallinity, and interchain attractive forces,^45^ and show glass transition temperature in the range of 90–174 °C for native, residual, and technical lignin.^47^ Molecular dynamics simulations can provide atomistic scale insight and thus connect the molecular structure of lignin to the predicted *T*_g_.

LeBoeuf and Zhang^48^ investigated the glass transition temperature of two lignin models (softwood and hardwood) using MD simulations with the COMPASS force field and by the broken-stick method. In the broken-stick approach, the equilibrated density, or equivalently the specific volume, of each lignin model is evaluated across a series of temperatures. Separate linear fits are applied to the low-temperature glassy regime and the high-temperature rubbery regime, and the intersection of the resulting linear fits is used to estimate the glass-transition temperature (*T*_g_). This approach is adopted because it is based on the fact that above *T*_g_, enhanced segmental mobility alters the thermal expansion behaviour of lignin, resulting in a distinct change in the slope of the specific-volume–temperature relationship, and it is easily implemented in MD simulations. Additionally, they have calculated the thermal expansion coefficient, density, and solubility parameters of the two lignin models. In their model, softwood lignin is composed only of G units and has a higher proportion of aliphatic–OH groups compared to phenolic–OH and methoxy groups. The hardwood model comprises both G and S units, and the methoxy groups are present in the highest amount in both G and S units, followed by aliphatic and phenolic–OH groups. It was concluded that the predicted *T*_g_, density, and solubility of softwood lignin are closely aligned with the experimental results, whereas the hardwood model exhibits an uncharacteristically low solubility parameter, suggesting that this particular model may be a poor representation of hardwood lignin. In their study, hardwood lignin has a higher *T*_g_ than softwood, which contradicts experimental observations. Several factors, such as molecular weight and branching, are also discussed in these models. In a recent study, Long *et al.*^49^ investigated by MD simulations the interaction between natural rubber and lignin (softwood and hardwood) using the Generalized Amber Force Field (GAFF) and reported that the simulated *T*_g_, density and mechanical properties predict well the experimentally measured properties. The primary driving force for lignin–lignin interactions was determined to be electrostatic forces, with contributions from hydrogen bonds and pi–pi stacking.

**Fig. 4:**
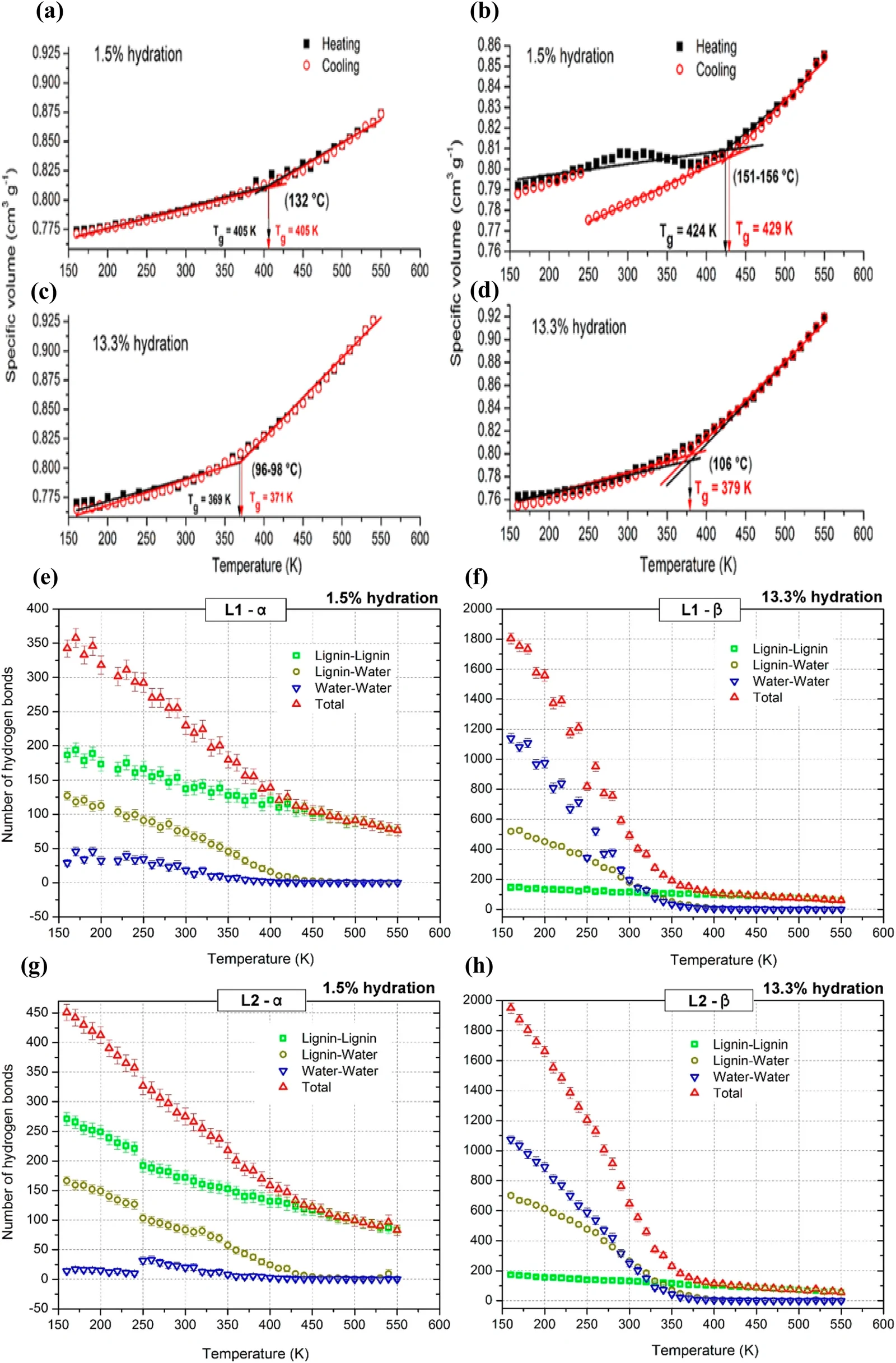
Curves of specific volume against temperature at (a) 1.5% L1-α (b) 13.3% L1-β for guaiacyl-rich lignin, (c) 1.5% L2-α (d) 13.3% L2-β for guaiacyl-type lignin. Number of hydrogen bonds with respect to temperature at (e) 1.5% L1-α (f) 13.3% L1-β for guaiacyl-rich lignin, (g) 1.5% L2-α (h) 13.3% L2-β for guaiacyl-type lignin. (Note: here, the total number of hydrogen bonds (HBN) has been obtained from the data on the cooling dynamics) (this figure has been reproduced from ref. 50 from American Chemical Society, copyright 2023).

The glass transition temperature is also dependent on the degree of hydration of the lignin network. Bregado *et al.*^50^ investigated the glass transition temperature of different lignin configurations by varying the hydration level, as shown in Fig. 4(a–d). Two lignin models were constructed: guaiacyl-type lignin (S/G = 0) (L2) and guaiacyl-rich lignin (L1) with S/G ratio of 0.38. They employed the Lignin-KMC software to generate two lignin types with low and high hydration concentrations, designated α and β, respectively (Fig. 4(e and f)). For the guaiacyl-type lignin, they used only 18G monomer units, whereas for the guaiacyl-rich lignin, they used 5S and 13G monomer units connected by various linkages. The simulations showed that *T*_g_ decreases with increased hydration; see Fig. 4(a–d). This behaviour is attributed to an increase in hydration, where the number of hydrogen bonds between lignin and water increases while the lignin–lignin H-bonds between phenolic hydroxyl groups decrease, as illustrated in Fig. 4(e–h). This fact can be attributed to increased hydration as water molecules replace direct lignin–lignin hydrogen bonds by preferentially forming new lignin–water hydrogen bonds, and they also increase the intermolecular spacing between lignin chains.

#### 3.1.2 Thermal stability and degradation

Understanding thermochemical conversion is crucial for lignin valorisation and usage as a sustainable source for chemicals. However, the thermal degradation of lignin is a complex process due to its numerous components and various decomposition pathways. In general, it exhibits a broad range of decomposition temperatures, as the structure comprises different oxygen functional groups that exhibit varying thermal stability. The thermal degradation of lignin is strongly dependent on its origin (softwood, hardwood, *etc.*), moisture content, and processing conditions, particularly temperature.^51^ In a recent study, Petridis *et al.*^52^ employed atomistic MD simulations to gain a molecular-level understanding of the structure, dynamics, and thermodynamic properties of softwood lignin with respect to temperature variation. They employed the CHARRM and TIP3P force fields for lignin and water, respectively, and concluded that with the increase of temperature, lignin undergoes a transition from compact conformations with glassy dynamics to extended polymer conformations with enhanced dynamics.

#### 3.1.3 Mechanical strength and elastic moduli

**Fig. 5:**
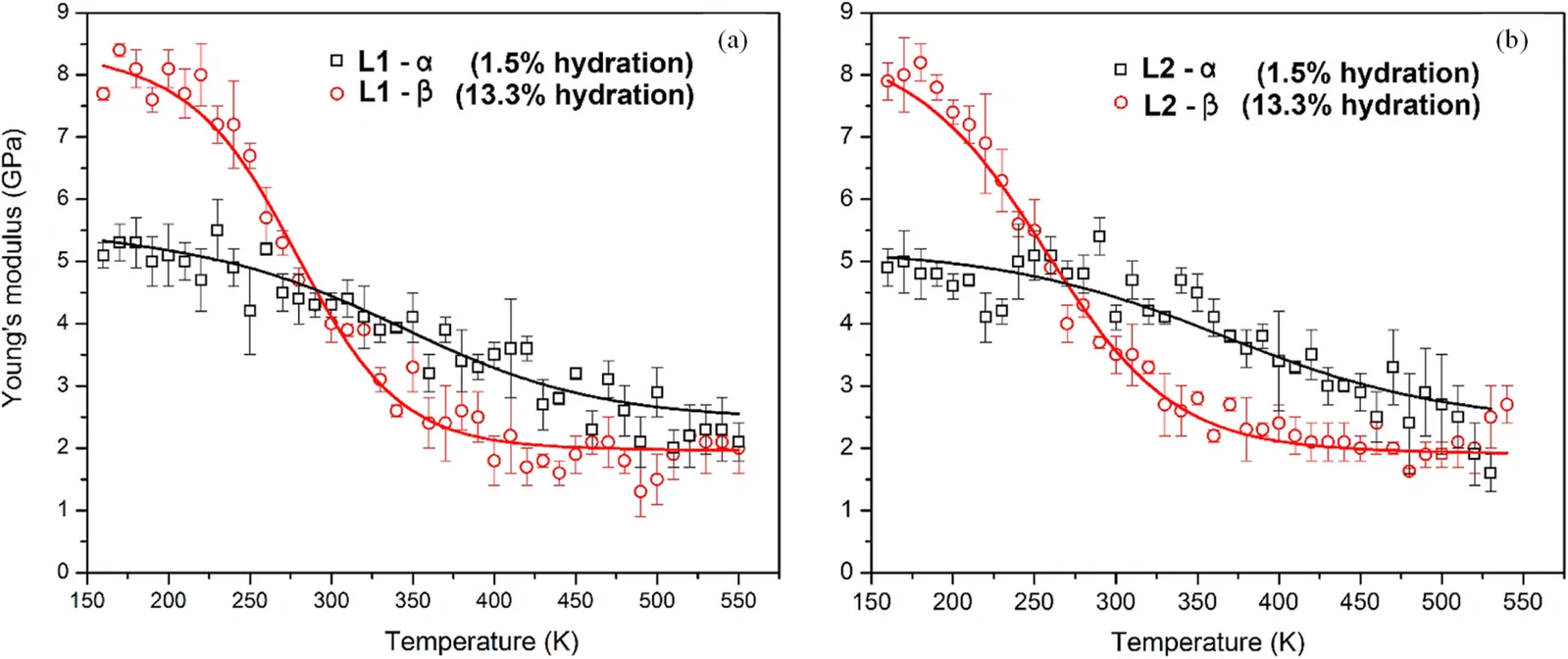
Plot of Young's modulus (*E*) with temperature (*T*) at 1 atm with two different hydration levels for (a) guaiacyl-rich (S/G = 0.38) lignin (b) guaiacyl-type (S/G = 0) lignin (this figure has been reproduced from ref. 50 from American Chemical Society, copyright 2023).

The lignin's mechanical properties are highly dependent on its chemical structure and level of hydration. Recently, Bregado *et al.*^50^ investigated the mechanical properties (Young's modulus and shear modulus) of softwood lignin at different hydration levels using MD simulations. Two types of lignin structure, guaiacyl-type lignin (S/G = 0) and guaiacyl-rich lignin with an S/G ratio of 0.38, were considered, and the temperature dependence of these properties is illustrated in Fig. 5. To calculate the Young's modulus, they performed simulations using the open-source software LAMMPS to calculate a stress–strain plot. They concluded that at lower temperatures, the mechanical properties of both lignin models with higher hydration (L1-β and L2-β) are better than those of the models with lower hydration (L1-α and L2-α). This is due to the fact that at lower temperatures with higher hydration, a large number of frozen or agglomerated water molecules are present, resulting in a higher stiffness (Young's modulus) of the lignin system. Additionally, from Fig. 4(f and h), it can be observed that at lower temperatures with a higher hydrated lignin system, the total number of hydrogen bonds (in red) is greater, resulting in better mechanical properties. Additionally, the calculated shear modulus of these lignin models at room temperature (300 K) was in good agreement with experimentally determined values for different lignin types.

### 3.2 Solubility and interfacial behaviour

This subsection highlights studies based on atomistic simulations to elucidate the lignin dissolution and self-assembly mechanisms, interactions with different polymers, and behaviour in various solvents. Understanding solvent interactions, self-assembly, and morphology across different solvents is beneficial for producing particles with desirable, tunable properties, such as particle size and shape. Combining simulation approaches with experiments is a viable way to elucidate solvation mechanisms and lignin self-assembly in different solvents.

#### 3.2.1 Lignin–solvent interactions (solubilization or dissolution mechanism)

**Fig. 6:**
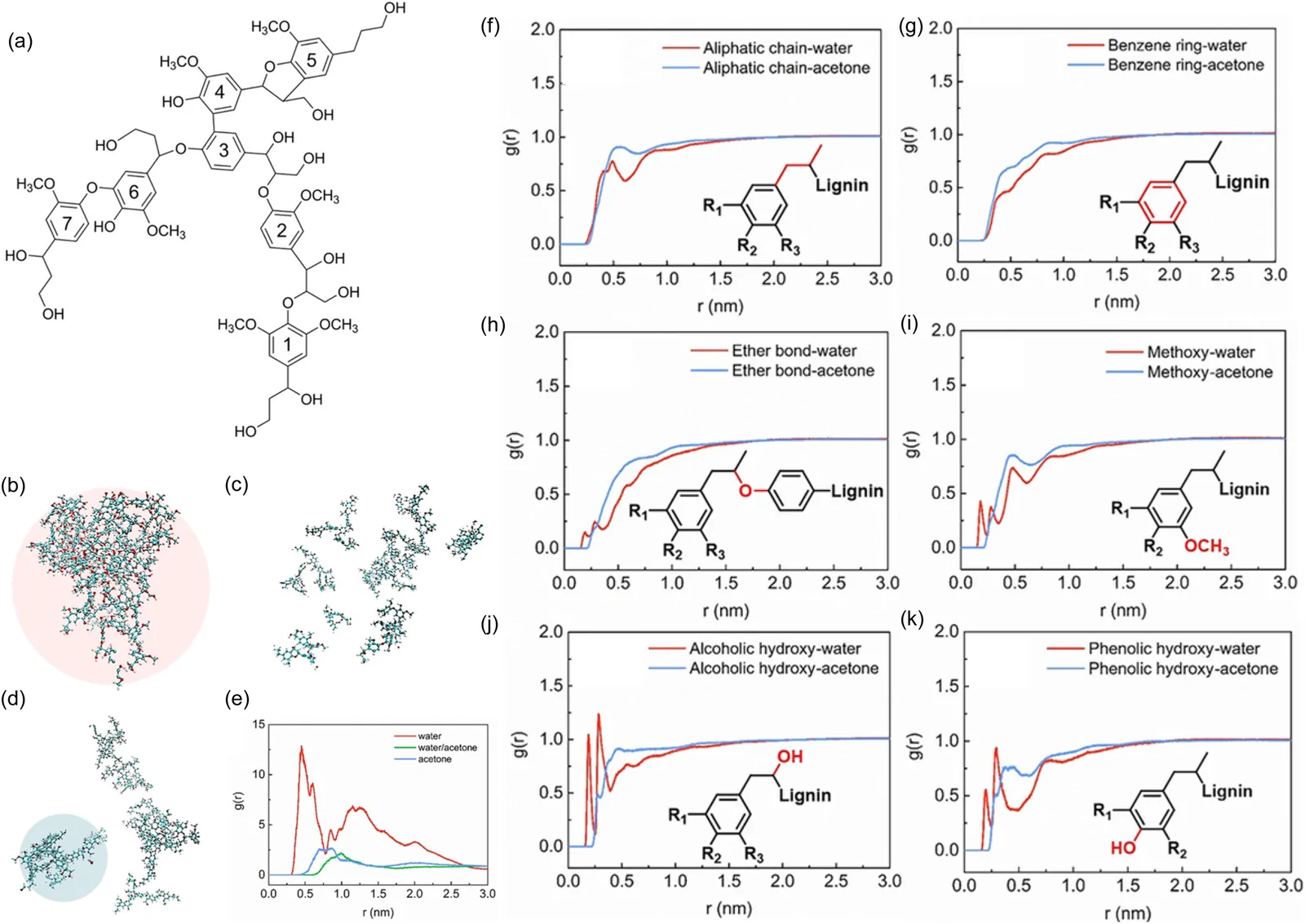
(a) Chemical structure of the lignin model used in the MD simulations. MD simulation snapshots of lignin self-assembly in (b) water, (c) acetone–water mixture (3 : 1), and (d) acetone. (e) Radial distribution function (RDF) of ring of lignin around water (red) and acetone (blue) and acetone–water solvent mixture (green); RDF of water (red) and acetone (blue) around different functional groups of the lignin model molecule in the acetone–water mixture solvent (f) aliphatic chain (g) benzene ring (h) ether bond (i) methoxy (j) alcohol hydroxy (k) phenolic hydroxy (note: all the corresponding functional groups of the lignin model are shown in red colour) (this figure has been reproduced from ref. 54 from John Wiley and Sons, copyright 2020).

Understanding the dissolution and aggregation behaviour of lignin is essential for accurately predicting its structure, size, and properties at the molecular level. Lignin is generally considered to be soluble in organic solvents rather than traditional inorganic solvents; however, some lignin oligomers are also soluble in water. Moreover, the dissolution behaviour of lignin also depends on the extraction and purification methods used. For example, kraft and soda lignin are soluble in aqueous alkali, while lignosulfonate is water-soluble.^37^ The dissolution behaviour of lignin is largely governed by intermolecular interactions such as hydrogen bonding and π–π stacking, as well as interactions between lignin and solvent molecules. Vu *et al.*^53^ have investigated the interaction between lignin and water using MD simulation with the PCFF force field. They have concluded that hydrogen bonds are responsible for reducing the mobility of water molecules in the vicinity of the hydroxyl and methoxy groups of lignin, as water molecules begin to structure around these groups. Wang *et al.*^54^ have provided insight into the aggregation and dissolution mechanism of enzymatic hydrolysis lignin (EHL) in three different binary organic solvents (acetone–water, tetrahydrofuran–water, and ethanol–water) at the molecular level with the use of atomic force microscopy and MD simulations to prepare lignin-based nanomaterials with controlled size and structure. Atomic force microscopy (AFM) provided a quantitative analysis of the dissolution mechanism, while MD-based simulations highlighted the molecular-level forces or mechanisms responsible for it in the acetone–water mixture. The lignin model consists of seven phenylpropane units with α-O-4, β-O-4, 5-O-4, 5–5, and β-5 bond linkages, as shown in Fig. 6(a). MD simulations of lignin in different solutions, including water, acetone, and a water–acetone mixture, revealed that lignin dissolves better in the water–acetone mixture (acetone : water ratio of 3 : 1) than in water or acetone alone, as shown in the Fig. 6(b–d). Fig. 6(b) shows that lignin strongly aggregates and forms larger clusters in water, while it aggregates moderately and forms smaller clusters in acetone (Fig. 6(d)), and disperses well in the acetone–water mixture (Fig. 6(c)). This solvent-dependent behaviour is further supported by the lignin–lignin (benzene rings from lignin) radial distribution functions shown in Fig. 6(e). The high and broad peaks obtained in water demonstrate a substantially increased probability of finding lignin molecules at short and intermediate intermolecular distances, confirming strong lignin–lignin association, while the lower RDF intensities in acetone and the acetone–water mixture indicate weaker intermolecular correlations and reduced aggregation.

The functional-group-specific RDFs in Fig. 6(f–k) provide a molecular explanation for the enhanced dispersion in the mixed solvent. Acetone exhibits a higher local density around the nonpolar aliphatic chains and benzene rings than water does, indicating its preferential solvation of the hydrophobic regions of lignin. Also, acetone shows favourable solvation of the ether- and methoxy-containing regions. The sharp short-distance peaks of water around the alcoholic and phenolic hydroxyl groups reflect strong hydrogen-bonding interactions with these hydrophilic sites. Thus, it can be concluded that the hydrophobic and hydrophilic groups of lignin are preferentially solvated by acetone and water, respectively. Consequently, by varying the acetone-to-water ratio, EHL-based lignin nanoparticles can be produced with controlled size and structure.

In general, lignin is poorly soluble in traditional solvents due to its strong intermolecular hydrogen bonding and π–π stacking. Ionic liquids are considered good solvents for cellulose as well as lignin.^34^ Zhu *et al.*^55^ reported a combined DFT and MD simulation-based study to investigate the molecular mechanism of dissolution of lignin (veratrylglycerol-β-guaiacyl ether (VG)) in imidazolium-based ionic liquid ([Amim]Cl). The authors employed the M06-2X functional with the 6-311+G(d,p) basis set implemented in the Gaussian 09 program package, while for MD calculation, the GROMACS package was employed with the AMBER force field. They concluded that, in most traditional molecular solvents, strong intramolecular hydrogen bonding and π–π stacking in lignin result in poor lignin solubility. Whereas in the case of ionic liquid, the dissolution of lignin is enhanced due to the fact that the cation and anion of [Amim]Cl show an important role in disrupting the intramolecular O–H⋯O–H-bonds of VG, and lead to better dissolution. In another study, the dissolution of macromolecular lignin in an ionic liquid-water cosolvent in 0%, 20%, 50%, 80%, and 100% 1-ethyl-3-methylimidazolium acetate (EmimOAc) was investigated at the molecular level.^56^ The improved solubility of lignin in solutions containing 50%, 80%, and 100% EmimOAc is attributed to the progressive disruption of interchain hydrogen bonds with increasing EmimOAc concentration. Janesko^57^ investigated the dissolution of lignin and lignocellulose in ionic liquid solvent using dispersion-corrected density functional theory (DFT-D), concluding that ionic liquid having π-conjugated cations favours the solubility of lignin. Furthermore, Vermaas *et al.*^27^ studied the interaction of lignin with different organic solvents like methanol, ethanol, tetrahydrofuran (THF), dimethyl sulfoxide (DMSO), acetone, 1,4-dioxane, and hexane using MD simulations. The authors employed GROMACS MD simulations on a lignin model consisting of β-O-4 linkages between guaiacyl monomers and solvated it in the aforementioned single solvents. Along with single-solvent analysis, they analysed results with mixtures of solvents such as THF/water (1 : 1) and dioxane/water (9 : 1). They concluded that there is no single solvent environment that is clearly superior to others in all cases, but DMSO is likely the optimum single solvent. A mixture of solvents can be tuned to have appropriate polarity, thus providing better solvating or solubilising features than single solvents. Apart from these atomistic and DFT-based studies, Zhu *et al.*^44^ proposed a mesoscopic study to understand the multilamellar spherical micelles of 2-chloroethyltrimethylammonium chloride (AC)-grafted alkali lignin (AL) using dissipative particle dynamics (DPD) simulations. They extensively studied the formation of lignin-based spherical micelles and examined the effects of AL content and molecular weight on the formation of multilamellar spherical micelles. They concluded that AL molecules can form spherical micelles in water, but AC-grafted modification of phenolic hydroxyl groups in AL (AC-g-AL) leads to the formation of multilamellar spherical micelles with an ordered structure. While changing the molecular weight of AL has no significant impact on the formation and sizes of multilamellar spherical micelles, it may affect the number of layers formed.

#### 3.2.2 Lignin–polymer blends and composites

Lignin is one of plants' main defence mechanisms against pests and contributes to the plant's structural integrity and strength, together with cellulose, hemicellulose, and other polysaccharides in the plant's secondary cell wall. It is challenging to explore lignin-polysaccharides interactions in intact cell walls, and most studies are performed on isolated materials, which significantly alter lignin structure during the pulping process. Therefore, computational-based studies are widely used to explore how lignin interacts with other polymers at the molecular level. In this subsection, lignin's interactions with natural polymers like cellulose and hemicellulose, as well as with synthetic ones, are summarised and discussed.

**Fig. 7:**
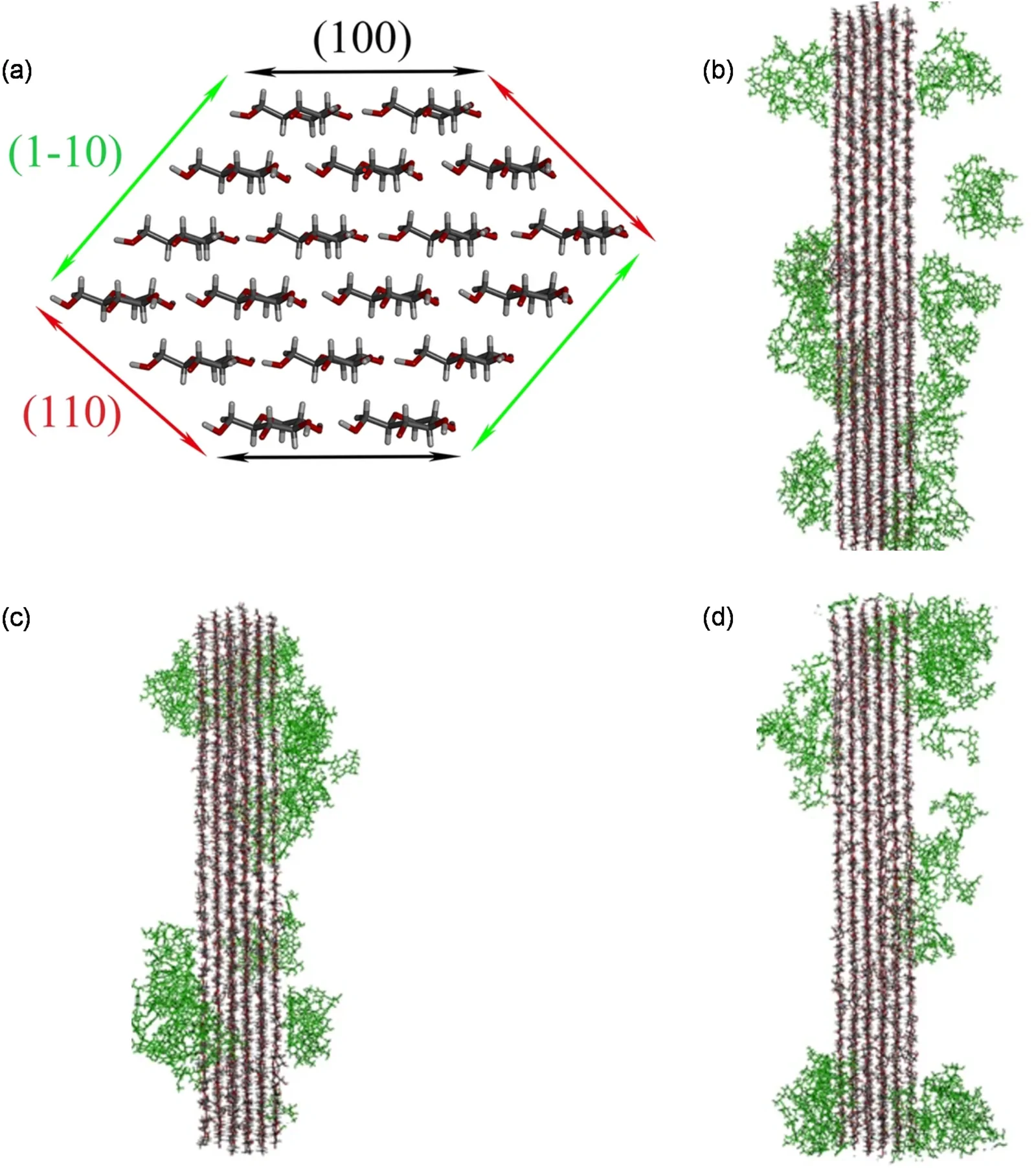
(a) A cellulose Iβ fibril cross-section highlighting the different cellulose surfaces. The (100) surface is indicated by the black arrows, the (110) surface in red, and (1–10) in green. (b) Snapshot showing confirmation of the cellulose fibril and lignin molecules, at (b) 300 K, (c) 400 K, (d) 550 K (this figure has been reproduced from ref. 59 with permission from Springer Nature, copyright 2022).

Vermaas *et al.*^58^ employed atomistic MD simulations to quantify the lignin monomers and dimers' interactions with different crystalline faces of cellulose. They concluded that lignin molecules bind most strongly to the more hydrophobic 100 surface, while among the hydrophilic surfaces, the affinity to 110 is the highest. Furthermore, the contact surface area between lignin and cellulose is found to be the most important factor in predicting the binding energy. Increasing the S/G ratio in lignin decreases the binding affinity due to the bulkier methoxy groups in the S-unit, which prevents the more favourable “flat” adsorption of the G-unit at the cellulose surface. Zhang *et al.*^59^ investigated the temperature-dependent interactions between lignin and a cellulose Iβ fibril (as shown in Fig. 7) using molecular dynamics simulations at 300, 400, and 550 K. They concluded that cellulose–lignin interaction can be controlled by altering cellulose surface hydration and lignin self-aggregation, as with increasing the temperature, the hydration layer surrounding the cellulose weakens, which allows a large accessible surface to lignin. Nair *et al.*^60^ investigated the wood swelling behaviour under moisture using MD simulations by varying lignin composition. They concluded that compression wood (CW) (enriched in 4-hydroxyphenyl units) has greater uniaxial swelling than opposite wood (OW) (enriched in guaiacyl units). The stronger expansion in CW comes from its greater phase separation from water and enhanced π–π stacking^61^ between aromatic units, whereas OW lignin forms more hydrogen bonds with water, resulting in more stable hydration and reduced swelling.

Long *et al.*^62^ investigated the effect of lignin type and concentration on the interactions between natural rubber (NR) and lignin. At lower lignin concentrations, highly branched hardwood lignin shows a weak interaction with NR due to lower electron density overlap with rubber chains. At higher lignin concentration, NR shows stronger interactions, as van der Waals interactions increase significantly due to higher atomic density and reduced polarity of hardwood lignin. Furthermore, the NR-lignin interactions are dominated by van der Waals forces, while dispersion and compatibility of lignin within the NR matrix are functions of both the type and quantity of lignin used.

#### 3.2.3 Self-assembly

Self-assembly is the process of spontaneous formation of an ordered structure driven by molecular interactions. Recently, lignin-based nanoparticles have attracted attention owing to their versatile functional properties, such as excellent dispersibility and UV-ageing resistance. These nanomaterials are generally formed by solution self-assembly in organic-aqueous (like acetone–water, tetrahydrofuran–water, and ethanol–water) solutions. For accurately controlling the size, structure, and properties of lignin nanoparticles, it is essential to know the self-assembly mechanisms. Two types of solvents are typically employed for the self-assembly: one with strong interaction with lignin and good solvating ability (good solvent), and the other with weak interaction with lignin and poor solvating ability (bad solvent). By adjusting the ratio of these two solvents, it becomes easy to produce lignin-based nanomaterials with precise structure, size, and properties. The self-assembly process also depends on the type of interactions between particles or molecular structures, such as electrostatic interactions, van der Waals forces, hydrogen bonding, and related non-covalent interactions. A recent review by Barrios *et al.*^63^ highlighted in detail the effects of these factors on the self-assembly mechanism of lignin. Nowadays, advances in molecular simulation make it easier to explore and elucidate the molecular mechanisms underlying lignin nanoparticle formation.

**Fig. 8:**
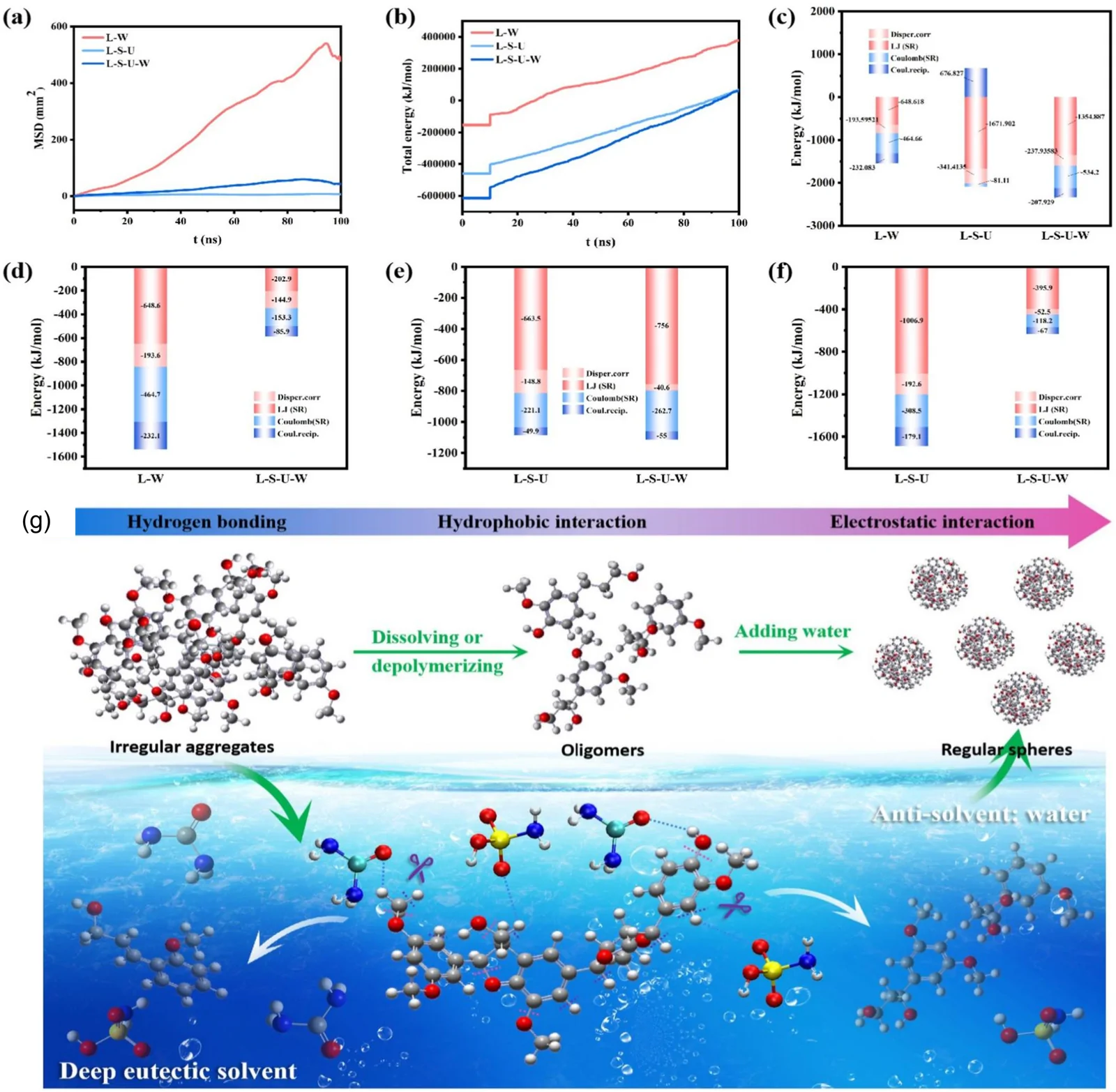
(a) Mean squared displacement of model lignin compound in different systems; (b) total energy of model lignin compound in different systems; (c) interaction energy of model lignin compound in different systems (d) interaction energy of model lignin compound with water; (e) interaction energy between model lignin compound and sulfamic acid; (f) interaction energy between model lignin compound and urea. (g) Mechanism of self-assembly of model lignin compound (this figure has been reproduced from ref. 64 with permission from Elsevier, copyright 2023).

To explain the self-assembly mechanism of the lignin nanoparticles, Wu *et al.*^64^ proposed a theoretical model based on quantum chemistry and molecular dynamics for lignin in a deep eutectic system. For this, they used the GROMACS MD package to run the simulation in combination with the OPLS-AA force field. The lignin structure was created with Gauss view by using 1H-type, 11G-type and 4S-type lignin structural units with 10 β-O-4, 3 C5–C5, 3 β–β, 2 β-5 and one 4-O-5 bond. They have calculated several physical parameters using MD simulations, including mean square displacement (shown in Fig. 8(a)), which reflects the diffusion coefficients of the lignin model, the total energy of the system (Fig. 8(b)), interaction energy (shown in Fig. 8(c–f)) (it reflects the intermolecular interaction and act as one of the driving factor for self-assembly), radius of gyration (it can give the details about how large space a molecule can occupy), number of hydrogen bonds, and radial distribution functions to explain the self-assembly mechanism of lignin. They concluded that hydrogen bonding, π–π interactions of aromatic groups within or between the molecules, and hydrophobic interactions are key factors responsible for the formation of LNPs. It was also shown that aromatic rings π–π interactions may occur in either a sandwich or T-shaped configuration.

In a recent article, Hackenstrass *et al.*^61^ investigated the π–π-stacking of simple lignin dimers, tetramers, and octamers composed of G units connected with β-O-4 linkages using molecular dynamics simulation in an aqueous system. To analyse π–π stacking, they employed three geometric criteria (distance, angle, and lateral displacement) and concluded that, across all developed models, intramolecular neighbouring rings have the greatest potential to form π–π stacking. For systems larger than a dimer, sandwich π–π stacking dominates, while intermolecular π–π stacking is rare in the studied systems, and T-shaped π–π stacking has little effect on the structural motif.

The self-assembly of lignin is a spontaneous, reversible process in which disordered lignin or nanoparticles assemble into structured materials due to the presence of non-covalent forces such as hydrogen bonding and π–π stacking, as shown in Fig. 8(g). From Fig. 8(g) it can be seen that the lignin molecules were initially dissolved and dispersed in the deep eutectic solvents. Further, it formed an oligomer due to the cleavage of the ether and carbon–carbon bonds. On addition of sulfamic acid, an amino group is formed, leading to more hydrogen bonds between the lignin's polar region and water and thereby increasing lignin's hydrophilicity. With further addition of the anti-solvent, the polar part of lignin tends to bind with water due to an increase in its degree of freedom. At the same time, the water molecules around the non-polar hydrophobic skeleton were repelled and extruded. This results in stabilisation of contact between two nonpolar regions, and the hydrophobic skeleton promotes π–π stacking and assembly into LNPs.

**Fig. 9:**
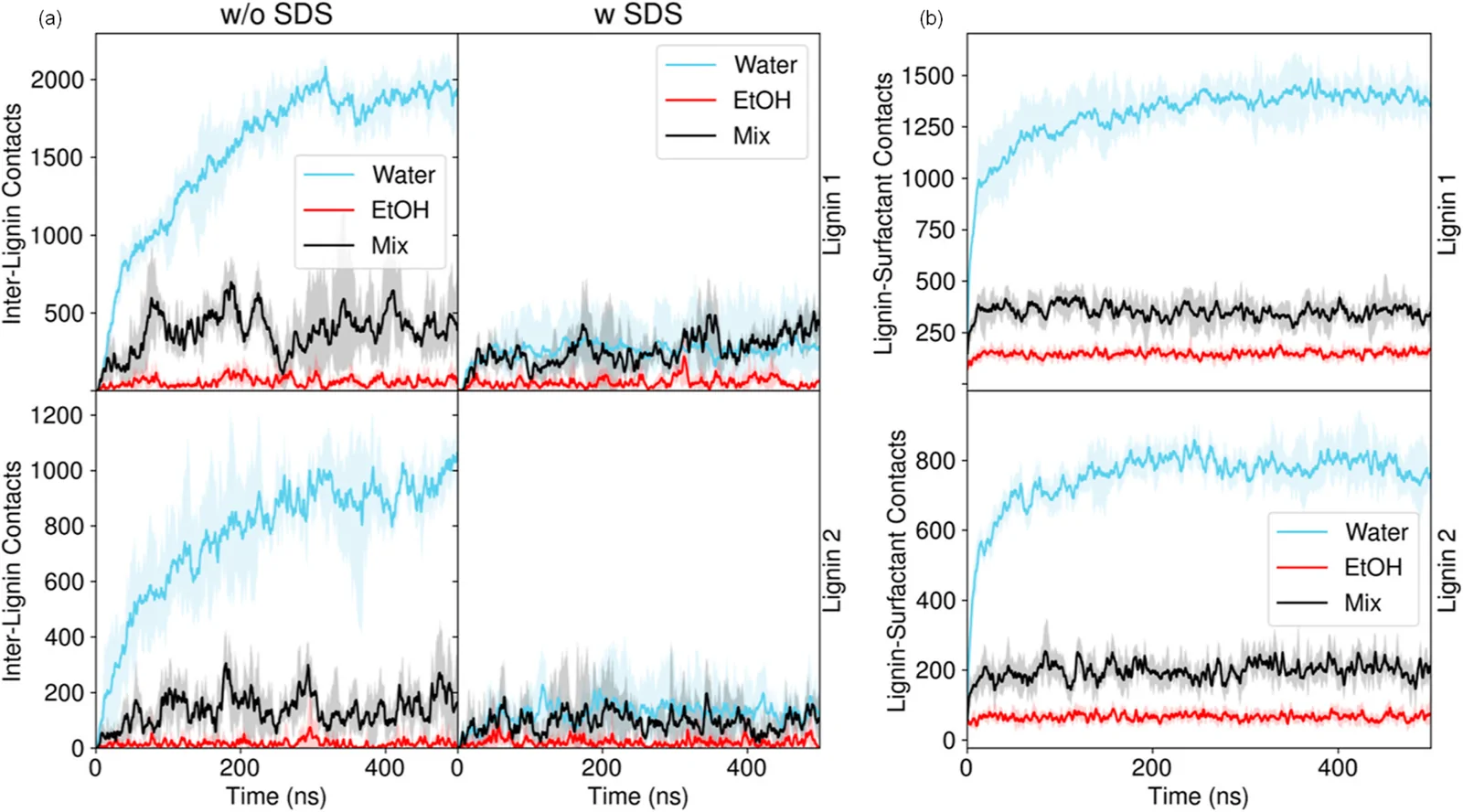
(a) Inter-lignin contacts during the simulation in water (blue), ethanol (95%) (red), and water/ethanol (32% ethanol) (black) with (right) and without (left) the surfactant SDS. (b) Lignin-SDS contacts during the simulation in water (blue), ethanol (95%) (red), and water/ethanol (32% ethanol) (black). The shaded areas represent the upper and lower bounds of the contact values observed across different simulations, and the darker line on the graph represents the average across the three simulation replicates (this figure has been reproduced from ref. 70 with permission from American Chemical Society, copyright 2022).

Earlier, it was reported that lignin molecules are amphiphilic in nature, and LNPs form *via* micellar or microemulsion processes.^65^ However, with advances in molecular simulation, the aggregation interactions and dissolution mechanisms of lignin in different solvents have been extensively studied, which has helped gain insight into the formation of LNPs.^66–69^ Recently, Ela *et al.*^70^ performed experiments and MD simulations to elucidate the formation and morphology of lignin nanoparticles in pure water, 32% and 95% ethanol, with and without the presence of the surfactant sodium dodecyl sulfate (SDS). They suggested that stable inter-lignin contacts form over time (as shown in Fig. 9) and improved lignin–solvent hydrogen bonding are among the driving factors behind the stable morphology. From Fig. 9(a), it can be seen that lignin in aqueous solvent has the most contacts between lignin polymers, while it is reduced on the addition of SDS, as most of the lignin contacts with water are mostly replaced by SDS in aqueous solvent (Fig. 9(b)). This reduction in inter-lignin contacts is due to SDS's lipophilic nature. The addition of the surfactant to the solution mitigates the hydrophobic collapse and chain aggregation.

**Fig. 10:**
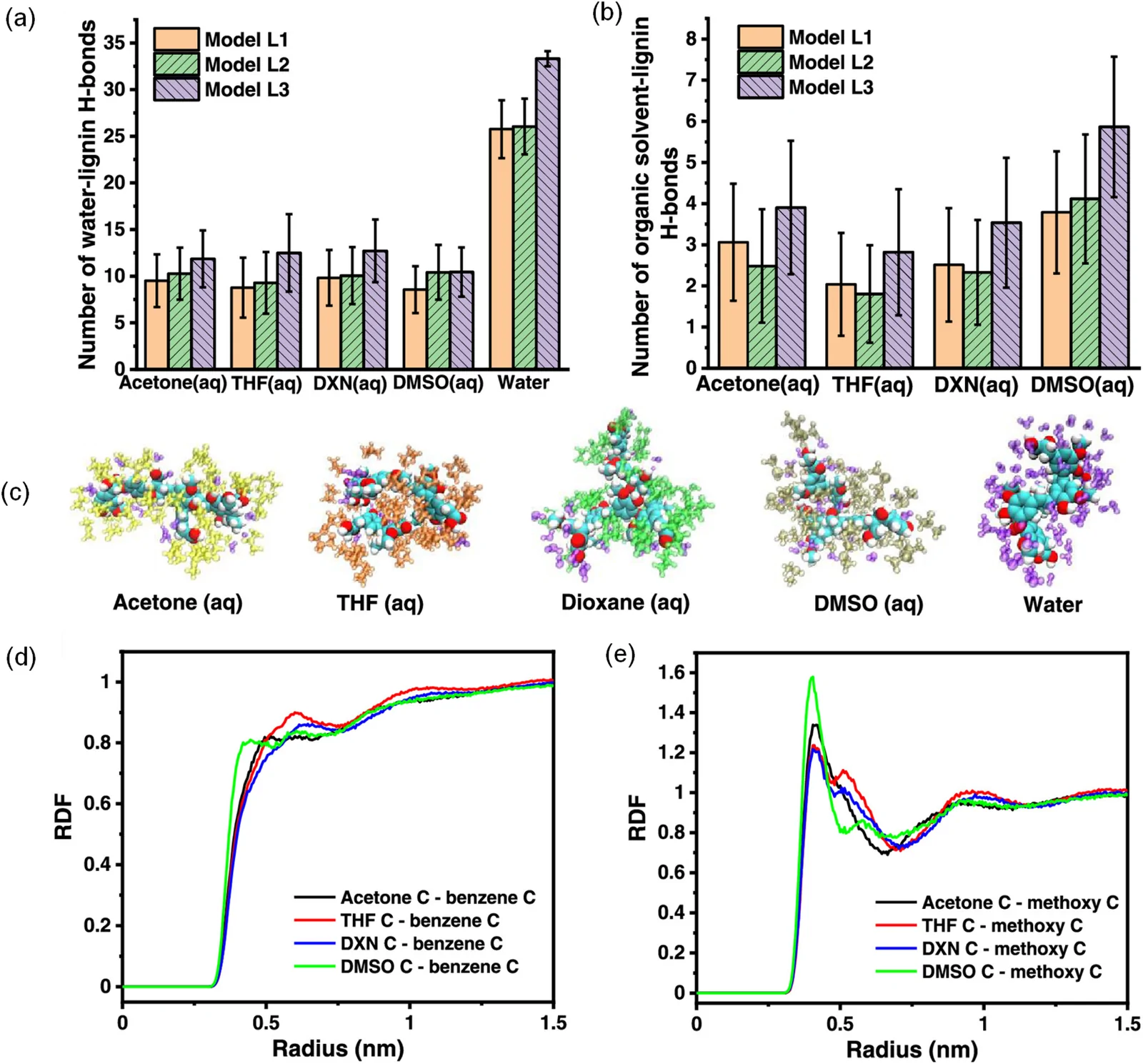
MD simulation data on the interactions between organic solvents/water and lignin models. (a) Number of H-bonds between water and lignin models in aqueous organic solvents (75 wt%) and water. (b) Number of H-bonds between organic solvents and lignin models in aqueous organic solvents (75 wt%). (c) Visualisation snapshots of the solvation of model L1 in different solvents showing displacement of water from the lignin solvation shell of 3 Å radius by organic solvents. Water is represented in purple in the visualisations, acetone in yellow, THF in brown, DXN in green, and DMSO in tan. (d and e) RDFs of the organic solvents around the hydrophobic moieties of the model L1 in the aqueous organic solvents (75 wt%). The RDFs are calculated using the C atom of the organic solvent and the C atom of (d) benzene or (e) methoxy group of the model L1 (this figure has been reproduced from ref. 32 under the terms of the Creative Commons Attribution-Non-commercial 4.0 License, American Chemical Society, copyright 2021).

In continuation of exploring the morphology of LNPs in different solvents, Zou *et al.*^32^ have investigated the effect of lignin–solvent interaction on the particle size of LNPs in binary solvents of aqueous acetone or aqueous tetrahydrofuran (THF) and pure water using MD simulations to complement the experimental results. Based on the MD findings, they stated that all lignin models have better solubility in binary solvents, while they show poor solubility in pure water. This is because organic solvents can also solvate the hydrophobic moieties of the lignin molecules, whereas water can only solvate the polar group, as shown by snapshots in Fig. 10(c). Further, to find the exact reason behind the variation in the size of LNP, they investigated the solvent–lignin H-bonds (Fig. 10(a and b)) and solvent–lignin hydrophobic interaction (Fig. 10(c and d)) and concluded that the stronger the solvent–lignin interactions, the smaller the size of LNP would be.

### 3.3 Electronic and optical properties

Lignin is a highly aromatic biopolymer with unique optical and electronic properties that provide potential for expanding its applications in advanced functional materials. Due to the presence of aromatic structure and phenolic hydroxyl group, lignin demonstrates absorption in the ultraviolet region and serves as a potential material in different applications such as UV shielding materials, optical sensing, and imaging.^71,72^ In this regard, computational approaches, particularly density functional theory (DFT) and time-dependent density functional theory (TD-DFT), have been widely used to investigate the electronic structure and optical behaviour of lignin model compounds and oligomers.

Durbeej and Eriksson^73^ investigated the spin distribution of unpaired electrons in dehydrogenated coniferyl alcohol and associated dilignol radicals, which provides insight into the radical stability, reactivity, and delocalization behaviour of lignin. They concluded that the majority of unpaired electrons are primarily delocalized over the phenoxy radical (O4) in both mono- and dilignol radicals. Kramer *et al.*^74^ employed DFT, quantum theory of atoms in molecules (QTAIM) and electron localisation function (ELF) to investigate the effect of composition of the phenylpropanoid units and oxidation of the α-hydroxyl group on the electronic stability and β-O-4 bonding properties of lignin dimers. For this, 18 β-O-4-linked lignin models were divided into two groups. The first group included 9 non-oxidised dimers formed from the different combinations of syringyl (S), guaiacyl (G), and *p*-hydroxyphenyl (H) units: SS, SG, SH, GS, GG, GH, HS, HG, and HH, while the second group consisted of nine structures in which the α-hydroxyl group was oxidised to an α-carbonyl group: SSOX, SGOX, SHOX, GSOX, GGOX, GHOX, HSOX, HGOX, and HHOX.

**Fig. 11:**
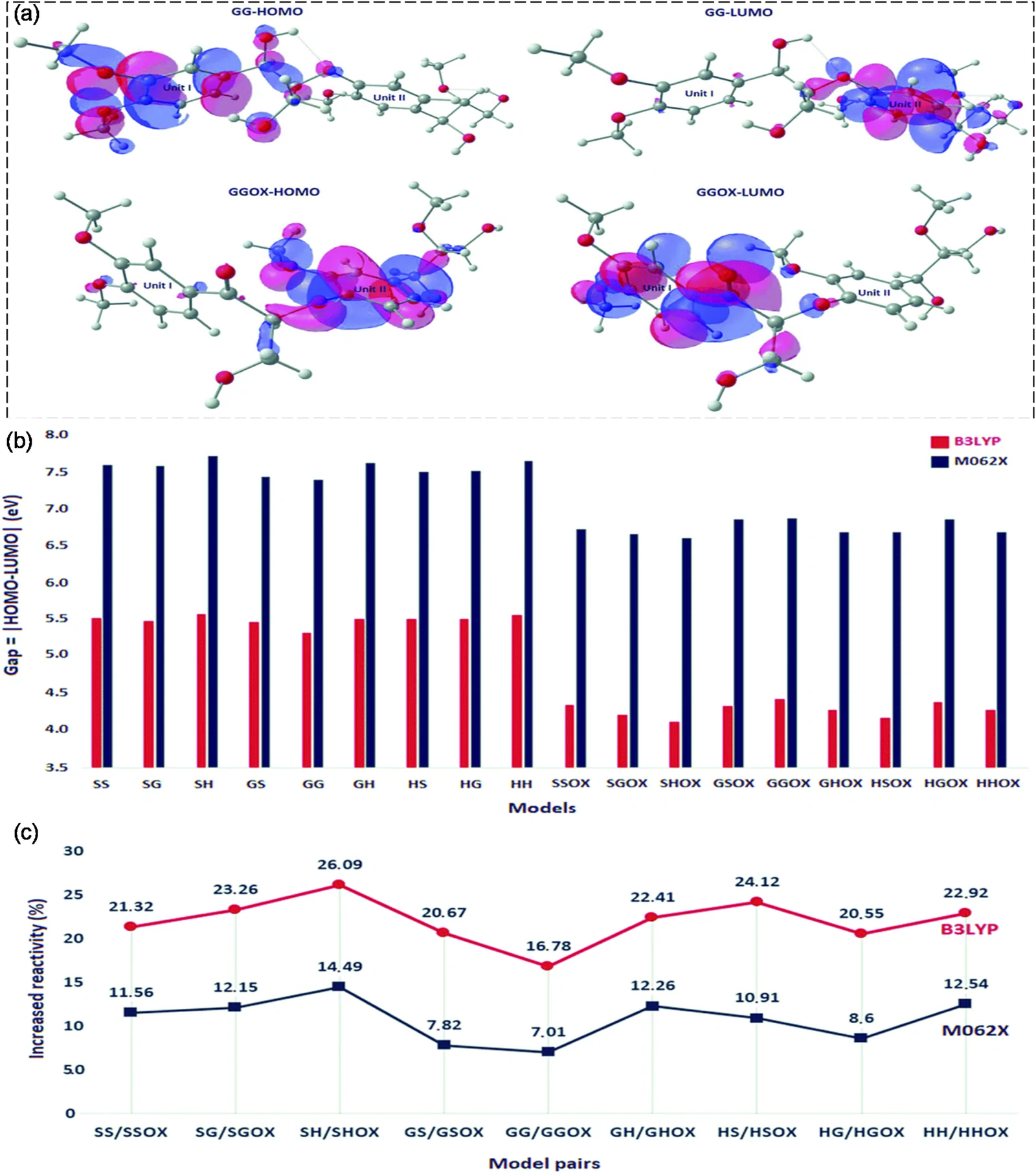
(a) HOMO and LUMO of the two different model GG (R_1_ = H; R_2_ = OCH_3_; R_3_ = H; R_4_ = OCH_3_) and GGOX (R_1_ = H; R_2_ = OCH_3_; R_3_ = H; R_4_ = OCH_3_); different lignin models with (b) HOMO–LUMO gap (c) value of reactivity (this figure has been reproduced from ref. 74 with permission from the Royal Society of Chemistry, copyright 2020).

They observed that the HOMO of the non-oxidised CG model is primarily located on unit I, having an α-hydroxyl-bearing side chain, whereas the LUMO is predominantly localised on unit II. However, for the oxidised GGOX model, the orbital distribution changed as the HOMO became localised mainly on unit II and the LUMO shifted towards the α-carbonyl-containing unit I (as shown in Fig. 11(a)). Thus, this redistribution demonstrates that α-carbon oxidation modifies the electronic communication between the two aromatic units. They also calculated the HOMO–LUMO energy gap as well as several electronic and structural descriptors of the β-O-4 linkage, including electron density, bond dissociation energy, and bond length as shown in Fig. 11(b). They concluded that among all the non-oxidised structures, SH exhibits the largest M06-2X energy gap, while SH and HH exhibit the highest or nearly highest B3LYP gaps, showing that these structures are among the most electronically stable and least reactive non-oxidised models. Among oxidised structures, GGOX was identified as the most electronically stable. They also investigated the percentage gain in reactivity (as shown in Fig. 11(c)) for each model and reported an increasing trend across all models and observed that oxidation of the alpha carbonyl is the crucial factor in the variation in stability. The maximum increase in reactivity was observed for the SH/SHOX pair, whereas the smallest increase occurred for the GG/GGOX pair.

Some studies have employed time-dependent density functional theory (TDDFT) to predict the optical properties of lignin.^75,76^ These studies demonstrate that lignin's optical properties are primarily governed by localised aromatic chromophores, methoxy substitution, and conformational flexibility. These studies demonstrated that the optical absorption of lignin chromophores is mainly governed by π–π* transitions associated with aromatic rings and n (non-bonding) π* transitions involving oxygen-containing functional groups such as phenolic and carbonyl groups. They also demonstrated that the presence of methoxy substituents and the degree of conjugation significantly influence the excitation energies and absorption wavelengths of lignin fragments. Syringyl units possess higher methoxy substitution and exhibit broader absorption and fluorescence than guaiacyl units.

### 3.4 Reactivity and functionalization

In recent years, lignin has emerged as a potentially sustainable and renewable resource for many applications. However, due to the complexity of its structure, the understanding of lignin conversion processes, such as catalytic depolymerisation, dissolution, pyrolysis, oxidation, and hydrogenation, remains limited. These conversion mechanisms are essential for several production processes and applications. Although a large body of experimental research exists on the conversion of plant biomass, studies specifically focused on lignin are comparatively scarce and often limited by the resolution of experimental methods. As a result, despite its tremendous potential for various value-added applications, lignin remains underutilised. Such experimental limitations have made the computer-based approach a viable tool for investigating lignin conversion. These approaches can provide pathways to understand the complex molecular-level transformation mechanism of lignin and, at the same time, offer insights to guide expensive experiments by providing a model for different conversion processes. This subsection focuses on various conversion pathways, such as dissolution, pyrolysis, and catalytic conversion, using MD and DFT-based computational approaches.

#### 3.4.1 Pyrolysis

**Fig. 12:**
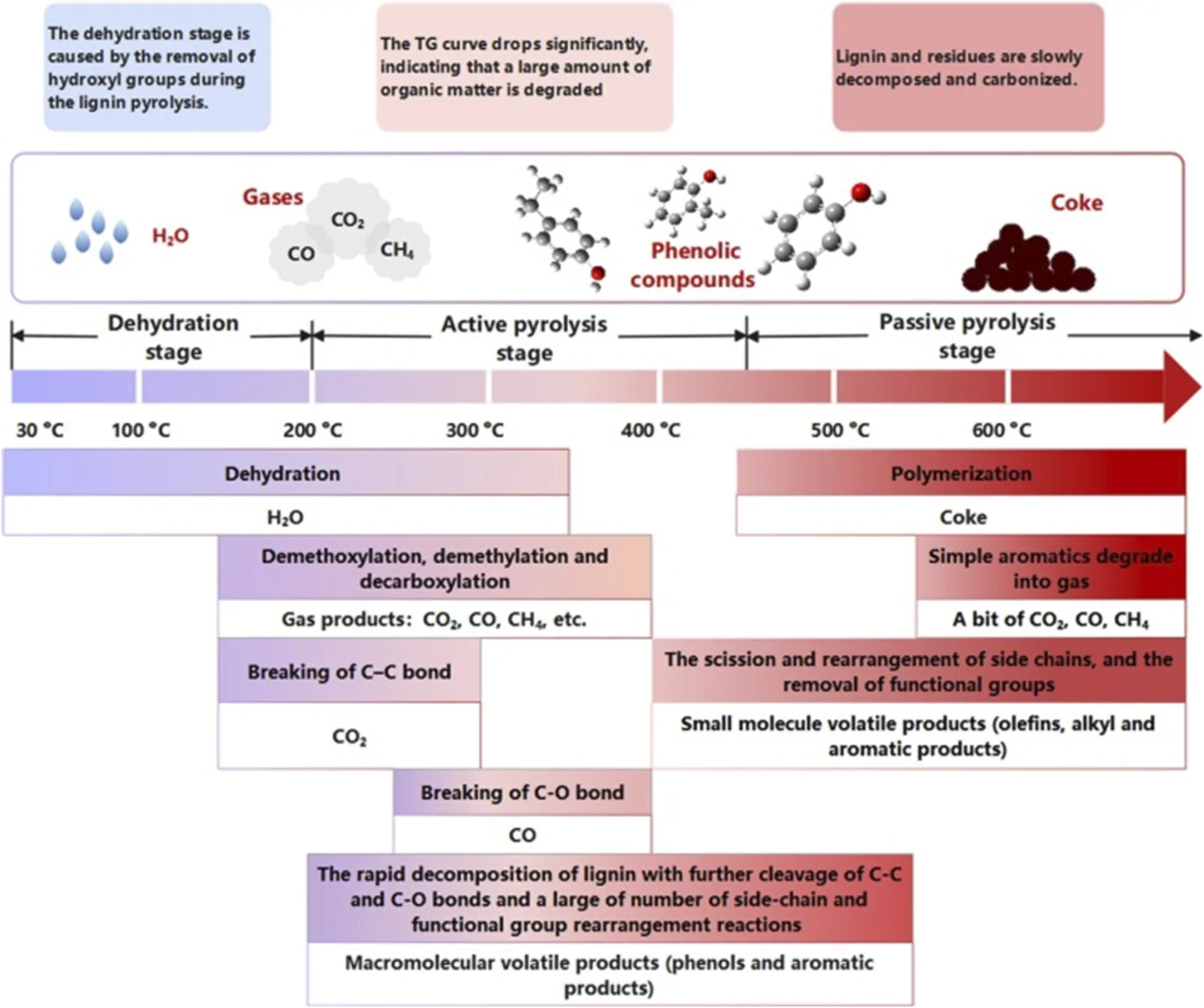
Scheme of lignin pyrolysis' different stages (this figure has been reproduced from ref. 84 with permission from Elsevier, copyright 2022).

Among all the available depolymerisation techniques, pyrolysis is one of the techniques that is used at high temperatures to break carbon–carbon (C–C) and carbon–oxygen (C–O) bonds.^77,78^ Due to lignin's complex random structure and the variation of many different linkages, the valorisation of lignin is challenging. The three different lignin monomers can react at 5 positions, yielding 7 possible bond types. The pyrolysis method, which is the thermal or thermochemical degradation of plant biomass in a controlled, small supply of air, has become an effective method for converting lignin into valuable liquid, gas, and solid products. In order to fully utilise lignin, understanding its pyrolysis mechanism is an important task. Although in the past few years, the pyrolysis mechanism of lignin has been extensively studied experimentally.^79–82^These studies are insufficient to provide detailed reaction pathways and mechanisms, because the complexity of lignin structure leads to a number of chemical reactions occurring on a sub-picosecond time scale. The pyrolysis mechanism of lignin can be divided into three stages: initial stage, primary stage, and the charring stage, as shown in Fig. 12. The initial stage involves dehydration and cleavage of weak bonds and the release of volatiles such as H_2_O, CO_2_, and CO in the temperature range of 160–330 °C. The primary stage, which occurs approximately in the temperature range of 330–600 °C, is responsible for extensive depolymerisation and fragmentation of β-O-4 ether bonds, producing phenolic monomers, guaiacol, syringol, catechol derivatives, and aromatic intermediates *via* radical reactions. The final charring stage occurs above 600 °C, responsible for secondary cracking, aromatisation, and repolymerisation, and results in the formation of stable aromatic hydrocarbons, as explained in detail for different types of lignin in previous articles.^81,83,84^

Recently, many papers have been devoted to predicting the reaction mechanisms during the pyrolysis of lignin. Younker *et al.*^85^ employed DFT calculations to investigate the bond dissociation energy (BDE) of C_α_–O and C_α_–C_β_ in β5 aryl coumaran lignin models using M06-2X/6-311++G(d, p) level theory and found that C_α_–O bonds show lower BDE (∼40–44 kcal mol^−1^), while C_α_–C_β_ bonds have significantly higher BDE (∼57–62 kcal mol^−1^), showing that ether bonds cleave more easily during thermal degradation. Huang and He^86^ employed DFT at B3LYP/6-31G(d,p) level of theory to explore the pyrolysis mechanism of the α-O-4 linkage lignin dimer model. They have also investigated the effect of alkali metal ions on the BDE, concluding that the homolytic cleavage reaction of C_α_–O is a major contributor, along with the homolytic cleavage reaction of O–CH_3_, and C_α_–C_β_. Huang *et al.*^87^ have investigated the detailed pyrolysis of the β-1 linkage in the lignin dimer using B3LYP/6-31G(d,p), focusing on the effect of temperature. They conclude that the BDEs of different bonds follow the following order: C_α_–C_β_ < C_β_–C_γ_ < C_α_–OH < C_α_–C_aromatic_ < C_β_–C_aromatic_ < C_aromatic_–OH, which means C_α_–C_β_ would be the first one undergoing bond dissociation. Also, the main components of the pyrolysis product are *p*-hydroxyphenyl-formaldehyde, *p*-hydroxyphenyl-methanol, *p*-hydroxyphenyl-acetaldehyde, and *p*-hydroxyphenyl-ethylene. There are a few more studies that provide a molecular-level picture, such as bond dissociation and reaction pathways, which are essential for selecting efficient lignin conversion strategies. DFT combined with kinetic modelling and pyrolysis experiments showed that the bond strengths and activation barriers of C–O is usually lower than C–C linkages. Also, β-O-4 bonds are the weakest among them and hence most reactive. In most of the lignin models, the homolytic bond cleavage of C_β_–O leads to the formation of a new product.^88–94^ It should be noted that BDEs are generally influenced by the computational methodology. Benchmark studies comparing a wide range of density functionals have shown that the choice of exchange–correlation functional generally has a much larger impact on the calculated absolute BDEs than the basis set, particularly for homolytic bond cleavage involving radical intermediates. In particular, M06-2X has been shown to provide more accurate and consistent BDE predictions than B3LYP across a benchmark of 17 density functionals, while increasing the basis set generally results in comparatively small changes in the calculated BDEs.^95^ However, the studies discussed here investigate different lignin linkage types and employ different molecular models and computational protocols. Consequently, a meaningful direct quantitative comparison of the reported BDEs or of the computational methods is not possible, and the results should instead be interpreted qualitatively within the context of each individual study.

**Fig. 13:**
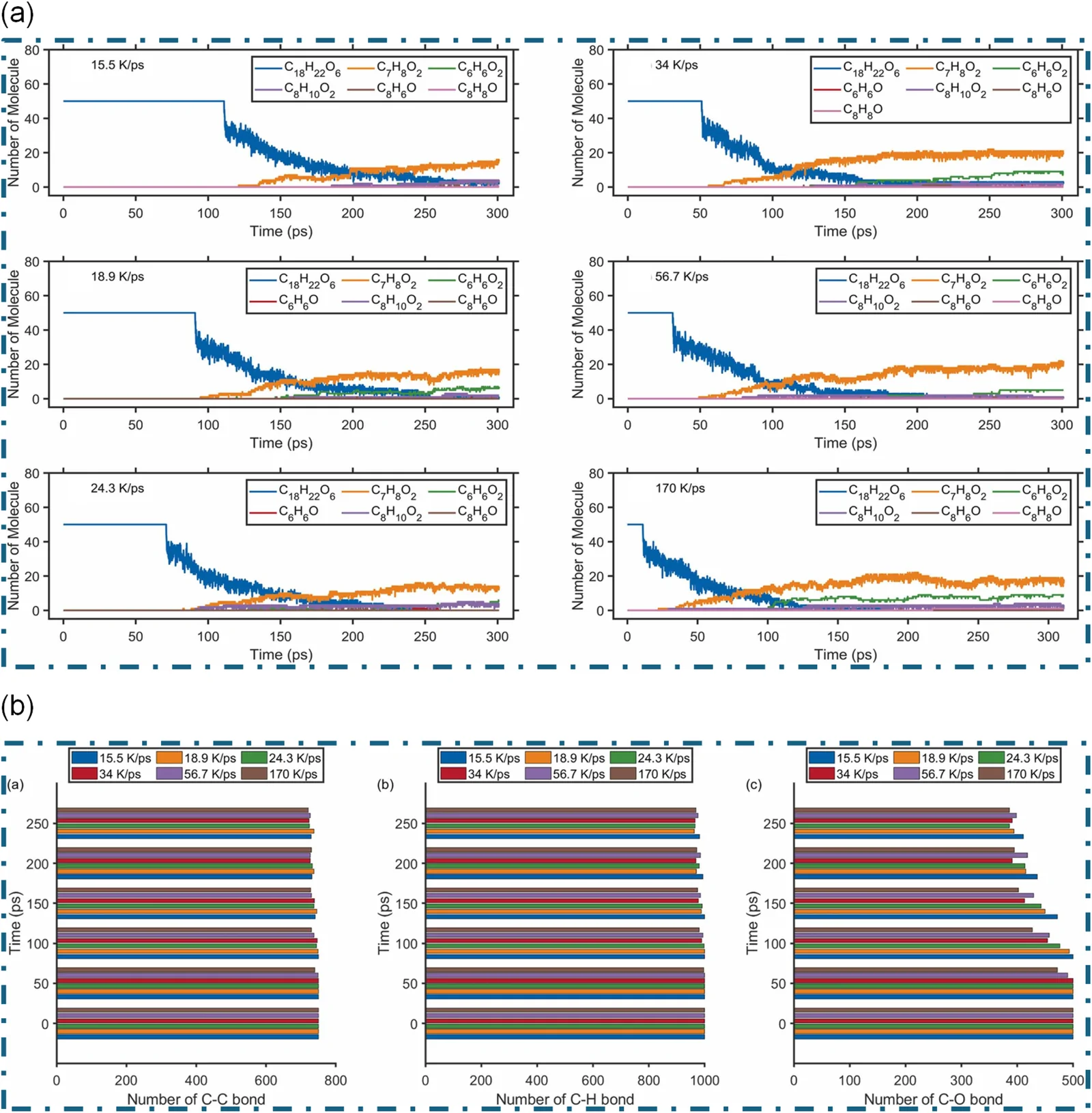
(a) Time evolution of major organic products at 2000 K with different heating rates (b) plot for time evolution of the total number of C–C, C–H, and C–O bonds at 2000 K with different heating rates during pyrolysis (this figure has been reproduced from ref. 101 with permission from Elsevier, copyright 2021).

Recently, new efforts have emerged in the field of investigation of the pyrolysis of lignin-based materials to study the larger lignin oligomers, utilising ReaxFF-based MD simulations.^96–100^ ReaxFF MD simulations are more efficient in terms of time and system size and yield results comparable to DFT studies, which is why they are employed to study the combustion and pyrolysis of lignin systems. ReaxFF is based on the bond-order concept to analyse bonds, interactions, and reaction mechanisms within complex systems.^96^ The accuracy and predictive capacity of ReaxFF mainly depend on the parameterisation based on DFT for calculating the parameters used in the force field. The ReaxFF potential modified by Han *et al.*^100^ can predict the BDE comparable to DFT and experimental results. Zhou *et al.*^101^ employed ReaxFF MD simulations to explore the effect of reaction temperature and heating rate by performing a number of simulations on the lignin dimer, namely 1-(3,5-dimethoxyphenyl)-2-(2-methoxyphenoxy) propane-1,3-diol (DMPD) with β-O-4 linkage by tracking the time evaluation of the product distribution and formation and breaking of chemical bonds. They have concluded that at higher temperatures, the tendency for the formation of CO and the transformation of guaiacol to catechol is greater. This fact can be attributed to the separation of the CH_3_ radical from guaiacol and its combination with catechol, which occurs simultaneously in the reaction. Also, a higher heating rate favours the generation of C5-12 (increased from 70.59% to 78.23%) and the breaking of the C–O bond. In Fig. 13(a), the major liquid products from the pyrolysis simulation at different heating rates are shown; it was found that guaiacol has the highest yield, followed by catechol, while the other products are either produced in lower quantities or consumed during the reaction. They also concluded that higher temperatures facilitate the cleavage of C–C, C–H, and especially C–O bonds, whereas the heating rate has an insignificant effect on bond cleavage and product evaluation (as shown in Fig. 13(b)).

**Fig. 14:**
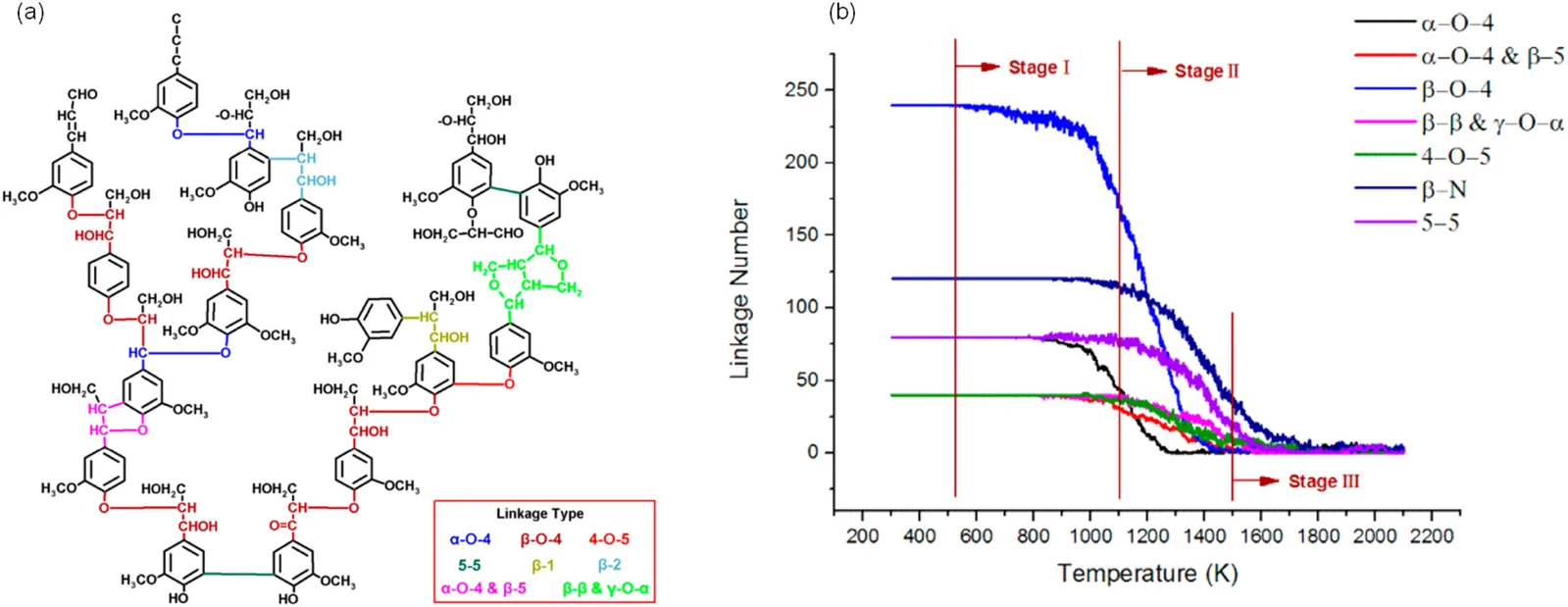
(a) Proposed model structure of spruce lignin by Adler, with different linkages annotated with colours; linkage evolution in the three stages of lignin pyrolysis obtained from the slow heat up (0.4 K ps^−1^) simulation of a large softwood lignin model with 15 920 atoms at 300–2100 K *via* ReaxFF MD, where β-N represents the linkages of β-1, β-2, and β-5: (b) linkage number (this figure has been reproduced from ref. 97 with permission from American Chemical Society, copyright 2017).

Zhang *et al.*^97^ have investigated the pyrolysis mechanism of a softwood lignin model (as shown in Fig. 14(a)) containing 15 920 atoms having different bond linkages using ReaxFF MD simulations. They performed the simulations using GMD-Reax, and the parameters used were taken from the Reax package in LAMMPS. Also, they employed the Visualisation and Analysis of Reactive Molecular Dynamics (VARxMD), which is a tool used to reveal the reaction information in the trajectory of ReaxFF MD simulation for analysing the internal relationship of atoms and their evaluation in the trajectory, to locate the linkage and its monomer-involved reaction by searching for the substructure in reactants or products. They concluded that the β-O-4 ether linkage breaks first under the temperature range 300–1500 K (as shown in Fig. 13(b)) and undergoes radical formation and chain reaction. Fig. 14(b) shows the three stages of linkage evolution in terms of the linkage number. The first stage corresponds to the complete decomposition of lignin molecules at low temperature, driven by the breaking of α-O-4 and β-O-4 linkages. In the second stage, the temperature is relatively high, leading to cracking of almost all bonds, whereas in the third stage, almost all linkages are broken or consumed. Additionally, the phenyl rings in the monomer undergo some rearrangement at high temperature.

#### 3.4.2 Catalytic depolymerization (bond-cleavage/dissociation)

Catalytic conversion of lignin is one of the most efficient ways to depolymerise it into monomers or oligomers that can be further utilised to produce value-added chemicals and sustainable functional materials. Lignin contains a high abundance of β-O-4 type linkages, therefore most of the experimental and theoretical studies have been conducted to investigate the corresponding reaction behaviour and mechanism. The advancement in computer simulation and the application of novel catalysts have facilitated a better understanding of lignin depolymerization and catalytic conversion.^102^ In this section, we review the state of the art for lignin depolymerization.

**Fig. 15:**
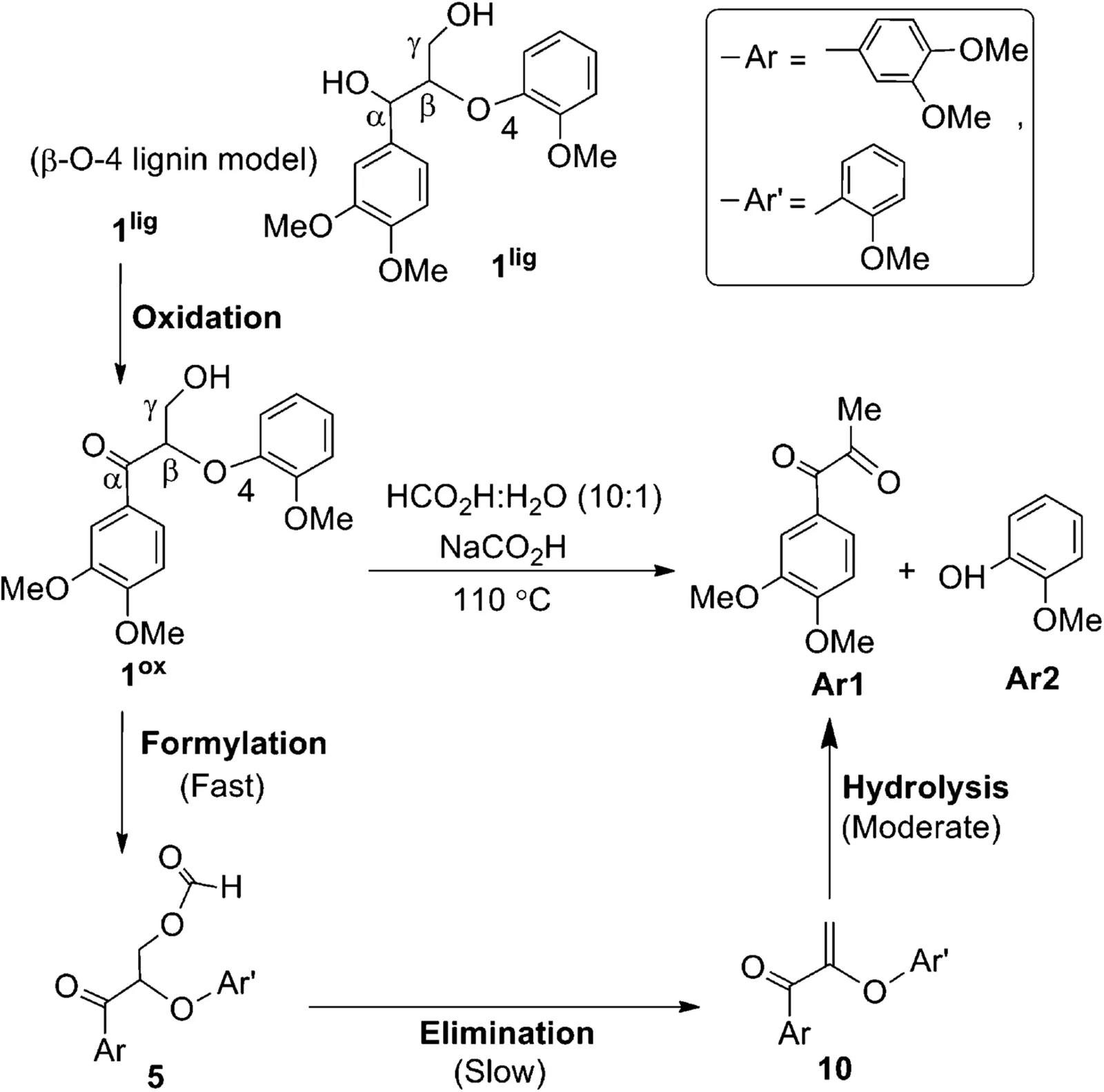
Flow chart of model depolymerisation of lignin to single aromatic compounds catalysed by formic acid (this figure has been reproduced from ref. 104 with permission from American Chemical Society, copyright 2015).

Chen *et al.*^103^ employed DFT computations to unravel the effect of the acid's H protons derived from the solid acid catalyst on the catalytic process of β-O-4 type lignin dimer. They conclude that the C_β_–O bond cleavage is responsible for the macromolecular breakage because the attack of H protons weakens the C_β_–O bond. Qu *et al.*^104^ studied formic acid-catalysed depolymerisation of lignin models to monoaromatics. The entire conversion process includes formylation, elimination, and hydrolysis, as illustrated in Fig. 15. They concluded that formic acid plays a vital role in accelerating the transformation, and the study also unravels the new elimination mechanism (E1H-3c4e) unconventional elimination mechanism which is characterized by passing through an intermediate stabilized proton shared 3c–4e bond (HCOO^−^⋯H^+^⋯^−^O=C_α_) and by shifting the 3c–4e HCOO^−^⋯H^+^⋯^−^O=C_α_ bond to the 3c–4e (HCO–O^−^⋯H^+^⋯^−^OOCH), which has a lower potential barrier compared to the earlier known E1cb and E2 mechanisms (E2 is a concerted elimination process in which H-abstraction and departure of the leaving group happen simultaneously, whereas E1cb is a stepwise elimination process that consists of two different transition states).

Ionic liquids also serve as effective reagents for the depolymerisation of lignin to obtain low-molecular-weight products. Singh *et al.*^105^ employed experiments and DFT to explore the depolymerisation of lignin using Brønsted acidic ionic liquid (BAIL, [C_3_SO_3_HMIM][HSO_4_]). Based on the DFT calculations, they found that weak and strong hydrogen bonding between the substrate and the ionic liquid significantly affects depolymerisation. In a recent study, Tao *et al.*^106^ performed a DFT study combined with ReaxFF MD simulations to explore the fundamental mechanism responsible for lignin gasification under supercritical fluid conditions (supercritical carbon dioxide (SCCO2) and supercritical water (SCW)), aiming to enhance biomass utilisation with reduced carbon emissions. For this, they built a lignin model in Materials Studio using 14 guaiacyl-based monomers, 1 *p*-hydroxyphenyl monomer, and 1 lilac monomer. Firstly, they performed a quantum-mechanical calculation in VASP to determine the lignin bond dissociation energy and the sequence of bond dissociation during gasification and then employed ReaxFF in LAMMPS to model the dynamic gasification process in both supercritical solvents. They concluded that in both environments, CO, CO_2_, H_2_O, and H_2_ formed as gaseous products. Also, the increase in temperature significantly increases the rate of product formation by enabling the attainment of dynamic equilibrium earlier. Moreover, DFT-based calculations can also provide an insight into the molecular mechanism of ether bond cleavage for different lignin models, and help tailoring the solvent or the catalyst for more effective catalytic depolymerisation of lignin systems. Ionic liquids or acidic media facilitate bond cleavage by lowering reaction barriers through proton transfer, hydrogen bonding, and catalytic anion effects, thereby enabling selective depolymerisation of lignin into valuable aromatic chemicals.^107–111^

Metals and metal oxides may affect lignin depolymerisation. Strassberger *et al.*^112^ employed DFT and experiments to identify the most suitable catalyst, which can cleave the β-O-4 bond while maintaining lignin's aromaticity. They concluded that copper particles loaded with γ-Al_2_O_3_ catalyse the hydrodeoxygenation (HDO) of β-O-4 lignin-type dimers and produce valuable aromatic products.

Stein *et al.*^113^ employed DFT and experimental methods and found that ruthenium can catalyse C–C bond cleavage in lignin, initiated by dehydrogenation. Cheng *et al.*^114^ employed DFT-based simulations to calculate the energy, geometry, and electronic structure of 1,2-diphenylethanol lignin on the (100) surface of three different metals (Ni, Cu, and W). They concluded that the presence of hydrogen affects the adsorption morphology, bond dissociation energy, and the stability of the C–C bond in lignin but does not affect adsorption on the W surface. Whereas the Ni surface interacts with the aromatic ring, the Cu surface cleaves the C–C bonds without the breakage of the aromatic ring.

Non-metallic catalysts have also shown a significant impact on lignin depolymerisation. Across various DFT-based catalysis studies, it has been shown that catalyst design and hydrogen delivery control C–O bond cleavage in some lignin-derived molecules. Bimetallic and metal catalyst (such as Ru–Ni, Pd) also tunes the adsorption strength and oxidation states for aryl ether and phenolic C–O bond linkage.^115^ DFT calculations have been instrumental in exploring the unique properties of metal catalysts in promoting the deoxygenation of lignin-derived phenolics.^116^ DFT calculations, in combination with experimental characterisation tools, can even explain C–C bond cleavage in lignin model compounds over a Nb-based catalyst (such as Nb_2_O_5_).^117^ In addition, investigations of hydrogenolysis of lignin-derived aryl ether under mild temperature and pressure conditions provide important insights for meeting fuel demands from biomass resources.^118^ For example, Kar *et al.*^119^ reported the hydrogenolysis of benzyl phenyl ether to phenol and toluene with the help of Pd nanoparticles at low temperature and low H_2_ pressure in a methanol and water medium.

### 3.5 Machine learning

Machine learning has emerged as an efficient alternative for predicting material behaviour rapidly with desirable accuracy. The development of ML interatomic potentials, including neural-network and Gaussian-process-based potentials, has enabled researchers to predict bond breaking and other lignin properties beyond the capabilities of classical force fields, such as CHARMM or OPLS, much faster and with accuracy comparable to quantum-mechanical calculations.^120^ ML-based models have helped researchers to predict the most susceptible lignin monomers' bonds for cleavage. Zhang *et al.*^121^ combined ML with DFT to predict the photocatalytic cleavage of the C–C bond in lignin structure, and concluded that the ML-based algorithm can predict the bond cleavage with approximately 99% accuracy. They also found that cleavage energy increases with an increase in the number of methoxy groups on the aromatic rings. Kim *et al.*^122^ combined an artificial neural network (ANN) with multiscale kinetic Monte Carlo (kMC) to investigate the reaction probabilities of lignin depolymerization. They concluded that integrating ANN with kMC enables real-time control of complex lignin properties without compromising accuracy, thereby reducing kMC's computational burden.

**Fig. 16:**
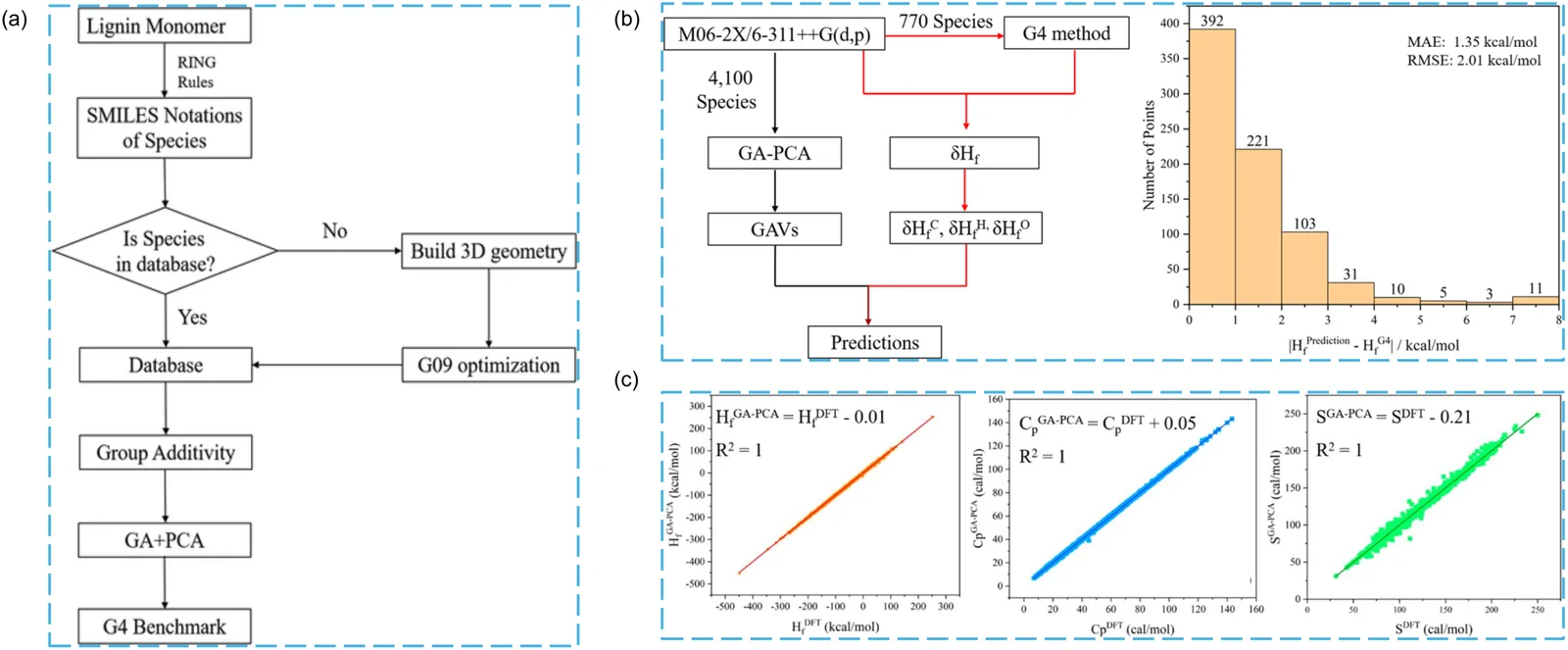
(a) Computational framework for database generation and group additivity model training. (b) DFT and GA-PCA predicted ((i) standard enthalpy of formation (Δ_f_*H*_298.15_), (ii) heat capacity (*C*_p_298.15__), and (iii) entropy (*S*_298.15_)) (c) strategy of correcting the GA-PCA model built at the M06-2X level to the G4 level and evaluation of predictions compared to the G4 subset (this figure has been reproduced from ref. 6 with permission from American Chemical Society, copyright 2021) (where, GA PCA stands for group additivity and principal component analysis; GAV-group additivity values).

Recently, Li *et al.*^6^ implemented machine learning, along with density functional theory and Group Additivity (GA), to study the steric and hydrogen-bonding effects of the substituent on bond dissociation energies and molecular geometry. For this, they first employed DFT-based computation to generate a database containing 4100 lignin-related molecules and radicals (as shown in Fig. 16(a)). The created database was used as input to the GA model, and its accuracy was enhanced by incorporating H-bonds and local steric effects (these effects were optimized using DFT calculation at the M06-2X/6-311++G(d,p) level), as well as a data fusion approach to improve the accuracy of the GA model to reach G4 theory of 770 species (as shown in Fig. 16(b)). The result plotted in Fig. 16(c) show the correlation between the predicted GA-PCA and DFT values for standard enthalpy of formation (Δ_f_*H*), heat capacity (*C*_p_), and entropy (*S*). From Fig. 16(c), it is clear that the GA-PCA model can readily reproduce the DFT-based data on bond dissociation energy (BDE) and the effects of substitutions.

**Fig. 17:**
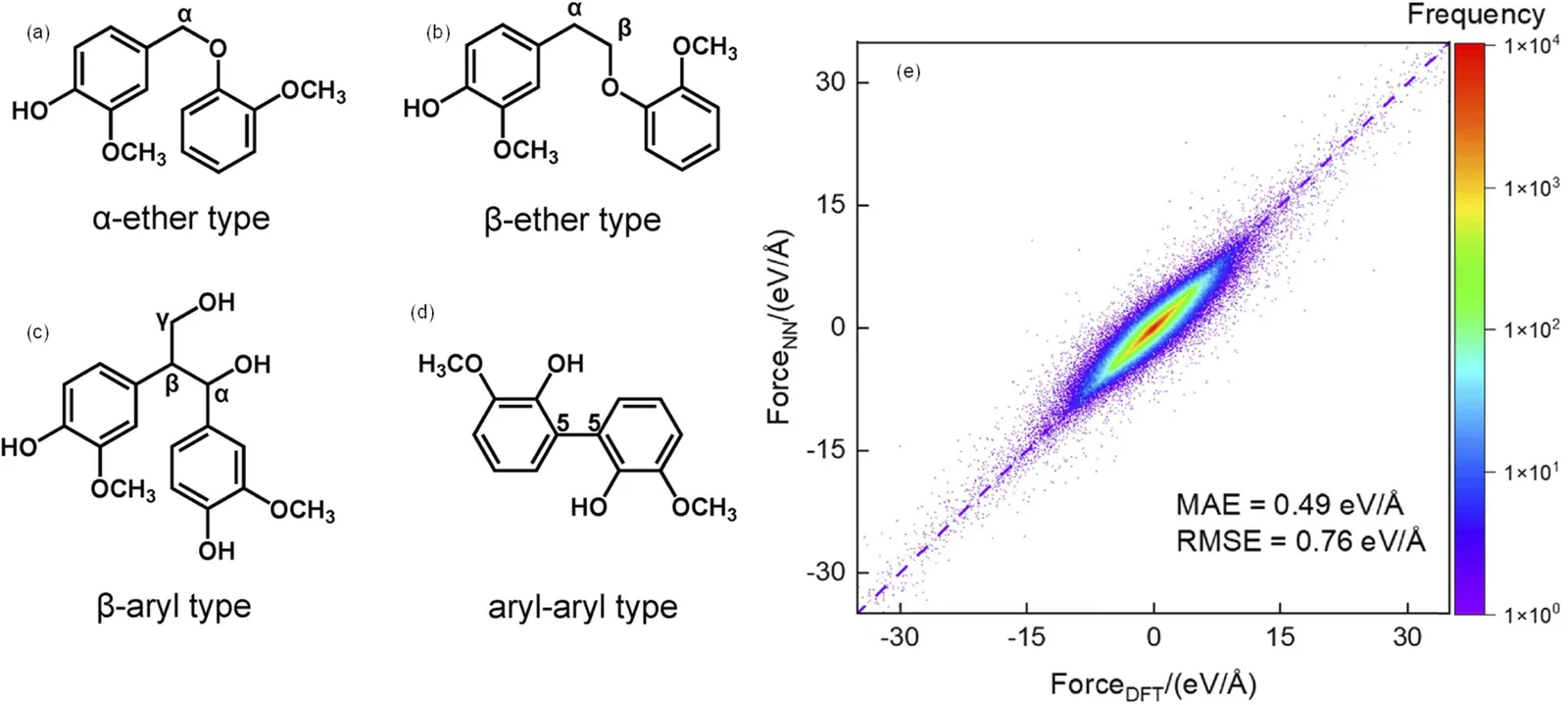
(a–d) Structure of lignin dimers. (e) The correlation between atomic forces predicted by the NN model and DFT (this figure has been reproduced from ref. 120 with permission from Elsevier, copyright 2024).

Recently, a neural network (NN) molecular dynamics simulation was employed to investigate the pyrolysis mechanism of lignin dimer.^120^ For this purpose, the data obtained from DFT were first used to train the NN force field, after which a series of MD simulations based on this force field were performed to explore the pyrolysis mechanism of four types of lignin dimers, see Fig. 17(a–d). The resulting NN force field demonstrated predictive accuracy closely matching the reference DFT values (as shown in Fig. 17(e)). They concluded that MD simulation with NN model can capture the entire pyrolysis process, which includes three different phases: (1) homolytic bond cleavage, which results in radical intermediates; (2) secondary transformation consisting of the removal of methoxy and the creation of gaseous/PHA; (3) ring-opening and aromatic coupling reactions, which result in complex species. The predicted atomic forces exhibited a Pearson correlation coefficient of 0.994 relative to the DFT reference values, with a mean absolute error (MAE) of 0.49 eV Å^−1^ and a root-mean-square error (RMSE) of 0.76 eV Å^−1^ (Fig. 16(e)). These results demonstrate that the developed model can overcome the limitations of quantum mechanical methods and classical molecular dynamics (MD) simulations in terms of computational efficiency and accuracy, respectively, thereby enabling the investigation of temperature-dependent pyrolysis pathways of lignin dimers.

## 4 Outlook and future perspectives

Despite significant advances in experimental and computational studies of lignin over the past decades, a comprehensive understanding of lignin structure–property relationships and transformation mechanisms remain incomplete. The intrinsic complexity of lignin, including its heterogeneous composition, irregular topology, and diverse bonding patterns, poses major challenges for both experimental characterisation and theoretical modelling. While computational approaches such as density functional theory (DFT), molecular dynamics (MD), reactive force fields, and machine learning models have provided valuable insights into lignin chemistry and material behaviour, several key aspects remain insufficiently understood and require further investigation.

One of the major challenges in computational studies of lignin is the accurate representation of realistic lignin structures. Most theoretical investigations rely on simplified model compounds including monomers, dimers, trimers, tetramers and other short oligomers that represent specific linkages, such as β-O-4, β-5, or β–β bonds. Although these models are useful for understanding fundamental reaction mechanisms, they often fail to capture the structural heterogeneity and polydispersity present in native and technical lignins. As a result, important macroscopic properties, such as thermophysical behaviour, mechanical properties, and solubility, may not be fully reproduced. Future computational studies should therefore focus on developing larger, more chemically realistic lignin models that reflect the variability in monomer composition, linkage distribution, molecular weight, and branching observed in natural systems.

Another major challenge concerns the prediction of thermophysical and mechanical properties of lignin. As discussed in Section 3, molecular dynamics simulations have been used to estimate properties such as glass transition temperature, density, mechanical moduli, and thermal stability. However, these predictions depend strongly on the accuracy of the employed force fields and on the representation of intermolecular interactions, such as hydrogen bonding and π–π stacking. Existing force fields, including CHARMM, OPLS, and GAFF, were originally developed for general organic molecules and often require additional parameterisation to accurately describe lignin-specific chemistry. Future work should therefore focus on developing and validating dedicated lignin force fields capable of describing a broader range of lignin structures and environments. Such improvements will enable more reliable predictions of lignin behaviour under realistic processing conditions. Understanding lignin dissolution, aggregation, and interfacial interactions remains incomplete. As discussed in Section 3, atomistic simulations have provided insight into lignin–solvent interactions and the mechanisms governing lignin solubility in organic solvents, aqueous mixtures, and ionic liquids. However, many simulations are still limited to relatively small systems and short time scales, making it difficult to capture long-range aggregation phenomena and solvent-dependent self-assembly processes. In particular, the formation of lignin nanoparticles and their morphology evolution involve collective molecular processes occurring on length scales beyond those accessible to conventional atomistic simulations. To overcome these limitations, multiscale modelling approaches combining atomistic simulations with coarse-grained molecular dynamics, dissipative particle dynamics (DPD), or mesoscale methods will be essential.

Coarse-grained simulations represent a promising strategy for investigating larger lignin systems and longer time scales. In such models, groups of atoms are represented as interaction beads, significantly reducing computational cost while retaining essential structural and interaction features. The recent development of the Martini 3 force field^123^ has opened new opportunities for coarse-grained modelling of complex biomacromolecules.^124^ Extending such frameworks to lignin systems could enable simulations of lignin aggregation, nanoparticle formation, and polymer–polymer interactions at mesoscopic scales. Further development and parameterisation of coarse-grained models specifically tailored for lignin will therefore play an important role in advancing computational lignin research.

Another important area requiring further investigation is the accurate computational description of the optical properties of lignin. As discussed in Section 3, quantum chemical approaches such as DFT and TD-DFT have been used to investigate the electronic structure and UV-visible absorption behaviour of lignin model compounds. However, TD-DFT studies of the optical spectra of realistic lignin macromolecules remain relatively scarce due to the large size (tens to hundreds of monomers) and structural heterogeneity of lignin. Future work should focus on extending quantum-chemical simulations toward larger oligomeric lignin structures and on integrating theoretical calculations with experimental spectroscopic techniques to achieve a more comprehensive understanding of lignin's optical behaviour. Such advances will be particularly important for emerging applications of lignin in UV-shielding materials, optical sensing, and organic electronic or photonic devices. Also, the computational cost of these methods limits their application to relatively small systems (hundreds of atoms). Future developments in linear-scaling quantum chemistry and fragment-based methods may enable more realistic modelling of larger lignin structures and their electronic properties. In particular, improved quantum-chemical descriptions will be important for understanding lignin's optical behaviour, photochemical reactivity, and potential applications in electronic and photonic materials.

Reactive simulations also represent an important area for future development. Methods such as ReaxFF molecular dynamics simulations enable the simulation of bond-breaking and bond-forming processes and have already been applied to study lignin pyrolysis and thermochemical conversion. However, the predictive accuracy of these models depends strongly on the quality of the training data used to parameterise the reactive force field. Expanding the available training datasets, particularly through high-level quantum-chemical calculations and experimental validation, will improve the reliability of reactive simulations of complex lignin systems. Such approaches could significantly advance the understanding of lignin conversion processes, including catalytic depolymerisation, gasification, and hydrodeoxygenation.

Machine learning techniques are also emerging as powerful tools for accelerating computational studies of lignin systems. Machine learning models can be used to predict thermodynamic properties, reaction energies, and bond dissociation energies at significantly lower computational cost than traditional quantum-mechanical calculations. Moreover, machine learning interatomic potentials, such as neural network or Gaussian process-based potentials, offer a promising approach to combining near-quantum accuracy with the computational efficiency required for large-scale molecular dynamics simulations. Integrating machine learning approaches with atomistic simulations and experimental datasets could significantly improve the predictive capabilities of computational lignin modelling. Another promising direction involves integrating machine learning with multiscale simulation frameworks. For example, machine-learning potentials trained on quantum-chemical calculations can be combined with molecular dynamics simulations to investigate larger lignin systems while retaining near-quantum accuracy. Similarly, data-driven models could be used to bridge atomistic simulations and mesoscale modelling approaches, enabling the prediction of lignin aggregation behaviour and nanoparticle self-assembly over experimentally relevant length and time scales.

Beyond predicting chemical reactivity, machine learning approaches could also play an important role in understanding the formation and properties of lignin-based nanomaterials, particularly lignin nanoparticles (LNPs). As discussed in Section 3, the formation of LNPs is governed by complex molecular interactions, including hydrogen bonding, π–π stacking, hydrophobic interactions, and solvent–lignin interactions. These processes involve large systems and long-time scales that are often difficult to capture using conventional atomistic simulations alone. Machine learning models trained on molecular simulation data or experimental datasets could therefore be used to identify key descriptors controlling lignin aggregation and nanoparticle formation. Such approaches may enable the prediction of LNP size, morphology, and stability as functions of solvent composition, lignin structure, and processing conditions. In this way, machine learning could assist in the rational design of lignin nanoparticle synthesis processes and accelerate the development of tailored lignin-based nanomaterials.

Overall, continued progress in computational modelling, including improved force fields, multiscale simulation strategies, reactive modelling approaches, and machine learning techniques, will play a crucial role in advancing the fundamental understanding of lignin. These developments will ultimately facilitate the rational design of lignin-based materials and enable more efficient valorisation of this abundant renewable resource in applications ranging from sustainable polymers and nanomaterials to energy and chemical production.

## Conflicts of interest

There are no conflicts to declare.

## Data Availability

No new experimental or computational datasets were generated in this review article. The data and results discussed are available from the original publications cited in the manuscript. Original schematic figures prepared for this review are included in the article. Literature-derived data used to prepare any original figures are provided in the article and/or are available from the corresponding author upon reasonable request. Figures or figure elements adapted or reproduced from previously published work are cited in the corresponding figure captions and used with appropriate permission where required.
